# Adipocyte fatty acid‐binding protein as a cerebrospinal fluid–accessible biomarker and druggable target in subarachnoid haemorrhage: Linking fatty acid dysregulation to microglial neuroinflammation

**DOI:** 10.1002/ctm2.70607

**Published:** 2026-01-30

**Authors:** Xingwu Liu, Shenquan Guo, Xin Feng, Hao Tian, Lei Jin, Boyang Wei, Wenchao Liu, Xin Zhang, Ran Li, Zhiyuan Zhu, Jingjing Kong, Xifeng Li, Lingling Shu, Chuanzhi Duan

**Affiliations:** ^1^ Neurosurgery Center Department of Cerebrovascular Surgery Engineering Technology Research Center of Education Ministry of China on Diagnosis and Treatment of Cerebrovascular Disease Zhujiang Hospital Southern Medical University Guangzhou China; ^2^ Neurosurgery Center Department of Functional Neurosurgery Zhujiang Hospital Southern Medical University Guangzhou China; ^3^ Clinical Biobank Center Zhujiang Hospital Southern Medical University Guangzhou China; ^4^ State Key Laboratory of Oncology in South China Collaborative Innovation Center for Cancer Medicine Sun Yat‐sen University Cancer Center Guangzhou China; ^5^ Department of Hematological Oncology Sun Yat‐sen University Cancer Center Guangzhou China; ^6^ State Key Laboratory of Pharmaceutical Biotechnology The University of Hong Kong Hong Kong China

**Keywords:** A‐FABP, CSF biomarker, fatty acid metabolism, microglia, neuroinflammation, neuroprotection, subarachnoid haemorrhage

## Abstract

**Background:**

Subarachnoid haemorrhage (SAH), a devastating subtype of stroke, is predominantly caused by the rupture of intracranial aneurysms. Emerging evidence indicates that the risk of intracranial aneurysm rupture correlates with elevated serum levels of fatty acids and pro‐inflammatory cytokines. Moreover, increased serum concentrations of adipocyte fatty acid‐binding protein (A‐FABP), an inflammation‐related adipokine, have been associated with poorer prognosis in SAH. However, the precise roles of A‐FABP in SAH pathogenesis and its biomarker potential in cerebrospinal fluid (CSF) remain unclear.

**Methods:**

CSF from 40 SAH patients and 30 controls was analysed by targeted fatty acid metabolomics. Experimental SAH mice were induced by endovascular perforation in both genetic deletion and pharmacological inhibition of A‐FABP. Brain injury was quantified by neurobehavioural test, inflammatory cytokine expression and TUNEL staining. In vitro, conditioned medium from fatty acid‐stimulated microglia was applied to primary neurons to evaluate apoptosis. Microglial metabolic reprogramming was assayed with Seahorse XF assays.

**Results:**

CSF revealed significant metabolic disruption in SAH, characterized by arachidonic acid (AA), linoleic acid and palmitic acid (PA). Enrichment analysis implicated A‐FABP plays a crucial role in SAH pathogenesis. Notably, elevated A‐FABP levels independently predicted increased SAH severity and poorer prognosis. In mice model of SAH, A‐FABP was significantly upregulated in microglia. Genetic deletion and pharmacological inhibition of A‐FABP significantly ameliorated brain injury, including neurological deficits, neuroinflammation and neuronal apoptosis. Mechanistically, PA and AA promoted BV2 microglial inflammation via an A‐FABP‐dependent manner, subsequently inducing apoptosis in co‐cultured primary neurons. Moreover, A‐FABP inhibition reprogrammed microglial metabolism, enhancing fatty acid β‐oxidation and energy supply. Proteomics further identified the JAK2/STAT3 as a downstream pathway of A‐FABP‐mediated neuroinflammation.

**Conclusions:**

A‐FABP is a promising biomarker and translatable therapeutic target to improve SAH outcome. Targeting A‐FABP disrupts fatty acids–driven neuroinflammation and microglial metabolic reprogramming to reduce brain injury after SAH.

## Highlights

- CSF A‐FABP levels predict disease severity and prognosis after subarachnoid haemorrhage.
- Genetic ablation or pharmacological inhibition of A‐FABP alleviates brain injury and improves outcomes.
- A‐FABP mediates fatty acid‐driven neuroinflammation in microglia via the JAK2/STAT3 pathway.
- Targeting A‐FABP reprograms microglial metabolism, enhancing fatty acid β‐oxidation and cellular energy supply.

## Graphical abstract

A‐FABP mediates neuroinflammation and exaggerates SAH outcomes: In response to SAH, elevated A‐FABP combines with fatty acids to promote microglia activation via the JAK2/STAT3 pathway, while simultaneously disrupting fatty acid metabolism. These dual effects exacerbate neuroinflammation and neurological deterioration, thereby contributing to adverse outcomes in experimental SAH models. However, when BMS interacts with the fatty acid‐binding pocket of A‐FABP, it inhibits the expression of pro‐inflammatory cytokines, thus attenuating neuroinflammatory responses and improving outcomes. Created with http://BioRender.com.

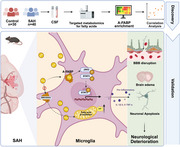

## 1 INTRODUCTION

Subarachnoid haemorrhage (SAH), a common subtype of stroke, is mainly caused by intracranial aneurysm rupture with high rates of disability and mortality.^1^ Early brain injury (EBI), the initial 3 days after SAH, is recognized as a major contributor to unfavourable outcomes.^2^ Targeting EBI represents a crucial therapeutic strategy for achieving a more favourable SAH prognosis. Accumulating evidence indicates that neuroinflammation is a central mechanism in EBI pathogenesis.^3^, ^4^ Thus, targeting the regulation of neuroinflammatory responses may hold therapeutic potential for improving outcomes in SAH patients.

Imbalanced fatty acid metabolism has been observed in various neuroinflammatory disorders and may serve as a critical driver of inflammation.^5^, ^6^ Existing evidence has indicated that elevated serum fatty acid levels (such as arachidonic acid [AA]), along with increased pro‐inflammatory cytokines, are closely associated with a higher risk of intracranial aneurysm rupture.^7^ However, the specific role of fatty acids in SAH following intracranial aneurysm rupture has yet to be fully explored.

Fatty acids, insoluble in water, require specific transport proteins, such as fatty acid‐binding proteins (FABPs). In addition to their conventional roles in fatty acid uptake and transport, FABPs also perform other functions in many processes, including inflammation, energy metabolism and cardiac dysfunction.^8^ Among the FABP family, A‐FABP is the most extensively investigated member. Although A‐FABP was initially identified in adipocytes, previous research has highlighted its deleterious role in macrophages.^9^, ^10^, ^11^

Furthermore, elevated serum A‐FABP has been identified as a predictor of unfavourable outcomes in patients with SAH.^12^ However, the underlying molecular mechanisms of A‐FABP in SAH remain to be fully elucidated. Unlike serum, cerebrospinal fluid (CSF) offers a more precise representation of the central nervous system's pathological state. Moreover, lumbar puncture to release CSF is a common procedure in the diagnosis and treatment of SAH. Yet, the expression and function of A‐FABP in the CSF of SAH patients remain unknown.

In this study, we demonstrated fatty acid metabolism disruption in CSF of SAH patients, with A‐FABP enrichment. Elevated levels of A‐FABP in CSF were found to predict SAH severity and poor outcomes. Pharmacological inhibition or genetic knockout (KO) of A‐FABP alleviated neurological deficits and brain injury in mice with SAH. Notably, A‐FABP, upon fatty acid binding, triggers microglia‐mediated neuroinflammation via activating JAK2/STAT3 signalling pathway. Furthermore, A‐FABP inhibition reprograms microglial metabolism, enhancing fatty acid β‐oxidation to ameliorate energy supply.

## 2 MATERIALS AND METHODS

### 2.1 Patients

Patients with aneurysmal SAH (aSAH) were consecutively enrolled from the Department of Cerebrovascular Surgery at Zhujiang Hospital (Ethics approval No. 2023‐KY‐280‐01). Control CSF samples were obtained from individuals receiving lumbar anaesthesia for non‐neurosurgical indications, who had no clinical evidence of neurological disease. Baseline clinical characteristics are provided in Table S1. Clinical severity was assessed at admission using the Hunt and Hess (H–H) grade. Functional outcomes were evaluated by neurological score modified Rankin Scale (mRS) at 3 months.

### 2.2 Biochemical and immunological analysis

CSF samples were collected once between Days 1 and 3 after SAH onset and stored at −80°C until assayed. The levels of A‐FABP were analysed using commercial enzyme‐linked immunosorbent assay kits. All the kits are from Jiangsu Meimian Industrial Co. Ltd.

### 2.3 Animals

Both male and female C57BL/6J mice (8–12 weeks) resided in controlled temperature and humidity conditions of Zhujiang Hospital (Project Number LAEC‐2023‐002). A‐FABP KO mice on a C57BL/6N background (generated as published protocol^13^) were matched with wild‐type (WT) littermate controls from the same heterozygous breeding pairs. Sample sizes per group were indicated in each figure legend. The experimental design is outlined in Figure S1.

Mice were gavaged orally with a selective A‐FABP inhibitor (BMS309403, #HY‐101903, MCE, USA; 15 mg/kg/day; dissolved in 4% Tween 80 and phosphate‐buffered saline) or vehicle (without BMS309403) one and 12 h after the sham or SAH surgery and then once per day for the next six consecutive days (concentration as previously described).^11^ JAK2 activator coumermycin A1 (C‐A1, #HY‐N7452, 100 µg/kg; MCE, USA) was administered intraperitoneally for 1 h after SAH.

### 2.4 SAH model

Mice were anaesthetized with 2% isoflurane (Matrx, #VMR) delivered via a calibrated nose cone vaporizer. Anaesthetic depth was maintained by monitoring the toe‐pinch withdrawal reflex and adjusting the isoflurane flow accordingly. SAH model was induced using the endovascular perforation as previously described.^14^ In brief, a monofilament suture (5‐0, polypropylene‐coated, 15 mm in length) was advanced from the left external carotid artery into the internal carotid artery until it reached and perforated the bifurcation of the anterior and middle cerebral arteries. The sham group received identical surgical exposure without vascular puncture step. Mice of both sexes were utilized in this study. A previously described grading system was applied to standardize SAH severity, with animals scoring below 8 excluded from the study.^14^

### 2.5 Open field test

Mice were placed in an open field consisting of a 50 cm×50 cm×40 cm plastic chamber. Each mouse was video recorded for 10 min. The following parameters were measured using Ethovision XT software (Noldus): total distance(cm) and total time(s). When evaluating the voluntary movement ability of animals, two movement states: static (less than 100 cm/s) and movement (over 100 cm/s) were observed. Fast movement proportion was calculated by movement time/total time.

### 2.6 Rotarod test

The Rotarod test (XR‐6C, Xinruan) was performed to assess motor function. Mice were pre‐trained for 3 days. The test procedure is as follows: started speed (4 rpm, acceleration time 5 s, duration time 60 s); primary speed (10 rpm, acceleration time 60 s, duration time 10 s); secondary speed (20 rpm, the acceleration time 60 s, duration time 10 s); and advanced speed (40 rpm, acceleration time 60 s, duration time 120 s). The variables recorded were the latency and the speed of the downfall. The data for each daily test were also presented with the average of three consecutive trials.

### 2.7 Brain water content

Brains were removed after anaesthesia. The wet weight of each brain was recorded immediately. Brains were oven‐dried for 48 h and re‐weighed to obtain the dry weight. Brain water content was then determined as [(wet weight − dry weight)/wet weight] × 100%.

### 2.8 TUNEL staining

TUNEL staining (terminal deoxynucleotidyl transferase dUTP nick‐end labelling kit; Beyotime, #C1086) was performed to evaluate neuronal apoptosis. Paraffin sections were stained with rabbit anti‐NeuN antibody and TUNEL assay kit. Images were acquired from the cortical region of the left hemisphere. The number of TUNEL‐positive nuclei merged with NeuN was analysed using ImageJ software (NIH).

### 2.9 Evans Blue staining

Blood–brain barrier (BBB) disruption was evaluated based on Evans Blue dye extravasation. The Evans Blue (Sigma‐Aldrich) was administered via intraperitoneal injection. Two hours after Evans Blue injection, mice were perfused with saline. Brains were then collected for imaging then spectrophotometric quantification of extravasated dye at 620 nm.

### 2.10 Immunohistochemical staining

The detailed methods regarding tissue processing, embedding and sectioning are described in our previous publications.^15^ Sections were blocked in 5% BSA and were incubated with the anti‐A‐FABP (.5 µg/mL, goat polyclonal; AF1443, R&D). The next day, immunohistochemical staining was performed using a staining kit (PV‐9000, Zhongshan Jinqiao Biotechnology Company) and diaminobenzidine (#9017, Zhongshan Jinqiao Biotechnology Company). Finally, sections were viewed under the 3D HISTECH Brightfield Slide Scanner (Pannoramic MIDI II).

### 2.11 Immunofluorescence staining

Paraffin‐embedded brain sections (4 µm) were blocked then incubated with the primary antibodies (Table S2). The next day, the slices were washed with PBS. They were incubated with the appropriate secondary antibodie: Alexa 647 or Alexa 488 (1:500, Invitrogen). Nuclei were stained with DAPI (G1012, Servicebio). Laser scanning confocal microscopy (Nikon, AX NIS‐Elements 5.4) was used to acquire a high‐resolution image. Images were acquired from the cortical region of the left hemisphere. The analysis was performed using ImageJ software.

### 2.12 Cell culture and fatty acid model

Mouse microglia cell line BV2 and mouse cerebral endothelial cell line bEnd.3 were obtained from the Central Laboratory, Southern Medical University. To mimic the fatty acid model in vitro, BV2 cells were treated with AA (#HY‐109590, 100 µM; MCE) and palmitic acid (PA, #P0500, 200 µM; Sigma‐Aldrich) as previously described.^16^

### 2.13 Primary cell culture

The cerebral cortex was isolated from P0 WT mice. Microdissection was performed to isolate the cortex by removing the meninges and basal ganglia. After collection, tissues were washed with PBS and digested with type IV collagenase and deoxyribonuclease I (#C8160‐100) at 37°C for 30 min. The cell suspension was filtered and centrifuged (1500 r/min, 10 min) and then plated on poly‐d‐lysine 6‐well plates. After 4 h, the supernatant was replaced with neurobasal medium containing .5 mM l‐glutamine and 2% B27 (Gibco).

### 2.14 Microglial culture supernatant transfer model

Primary cortex neuron cells were cultured in complete medium and treated with the supernatants of PA‐stimulated BV2 cells to test the neurotoxic effects of activated microglia. BV2 cells were pretreated to 200 µM PA ± 20 µM BMS for 24 h. Their supernatants were then collected, applied to primary cortical neurons for 12 h, followed by PBS wash and cell harvest for the next experiment.

### 2.15 Flow cytometry

Neuronal apoptosis in the BV2 culture supernatant transfer model was evaluated by flow cytometry (Elabscience Biotechnology). The apoptosis of primary cortex neuron cells harvested in the BV2 culture supernatant transfer model. For staining, cells were resuspended in 500 µL binding buffer containing 5 µL FITC‐Annexin V and 5 µL propidium iodide, followed by incubation at room temperature for 20 min. The number of apoptotic cells was assayed by flow cytometry (BECKMAN CytoFLEX). Data were processed using FlowJo software.

### 2.16 Western blot analysis

Western blot was performed as in the previous publication.^17^ The primary antibodies are provided in Table S2. Suitable secondary antibodies (1:10000, Proteintech) were selected. The bands were then observed using chemiluminescence reagent. The intensities were quantified using NIH ImageJ software.

### 2.17 RNA extraction and quantitative real‐time polymerase chain reaction (RT‐qPCR)

Total RNA was extracted using the EASY spin Plus RNA kit (RN28, Aidlab Biotechnologies). Total RNAs were reverse‐transcribed to cDNA with 5X Evo M‐MLV RT MasterMix (AG11706, Accurate Biotechnology). The cDNA was subjected to qPCR using SYBR Green‐based qPCR Kit (AG11701, Accurate Biotechnology). All reactions were performed in duplicate and normalized to the internal reference β‐actin for mRNAs. Primer sequences are listed in Table S3.

### 2.18 Seahorse XF assays

BV2 cells were plated onto XFe 96‐well culture plates (4000 cells/well) (Agilent Technologies) with PA (200 µM) and/or BMS (20 µM) stimulation for 24 h. Cells were washed and incubated in base medium (Agilent Technologies) at 37°C for 1 h. Glycolytic proton efflux rate (glycoPER) and oxygen consumption rate (OCR) were measured with Glycolytic Rate Assay and Mito Stress Test Kit, respectively, using the Seahorse XFe96 Analyser (Agilent Technologies). Data were analysed using Wave 2.6.1.

### 2.19 Determination of the ATP concentration

Following treatment with PA (200 µM) and/or BMS (20 µM) stimulation for 24 h, the BV2 cells were lysed, and the ATP content in the supernatant was measured with the ATP assay kit (Beyotime Biotechnology).

### 2.20 Statistical analysis

All data were presented as means ± standard deviation. Before each parametric test, we checked normality and equal variance. All statistical analyses were performed using GraphPad Prism 8 (GraphPad Software, Inc.). Two‐tailed *t*‐test and one‐way ANOVA were used for two groups and more than two groups, respectively. *p* < .05 was set as the criterion for statistical significance. Investigators were blinded to the identity of groups during the whole experiment.

## 3 RESULTS

### 3.1 Fatty acid metabolism is significantly disrupted in CSF of SAH patients, with A‐FABP enrichment

**FIGURE 1:**
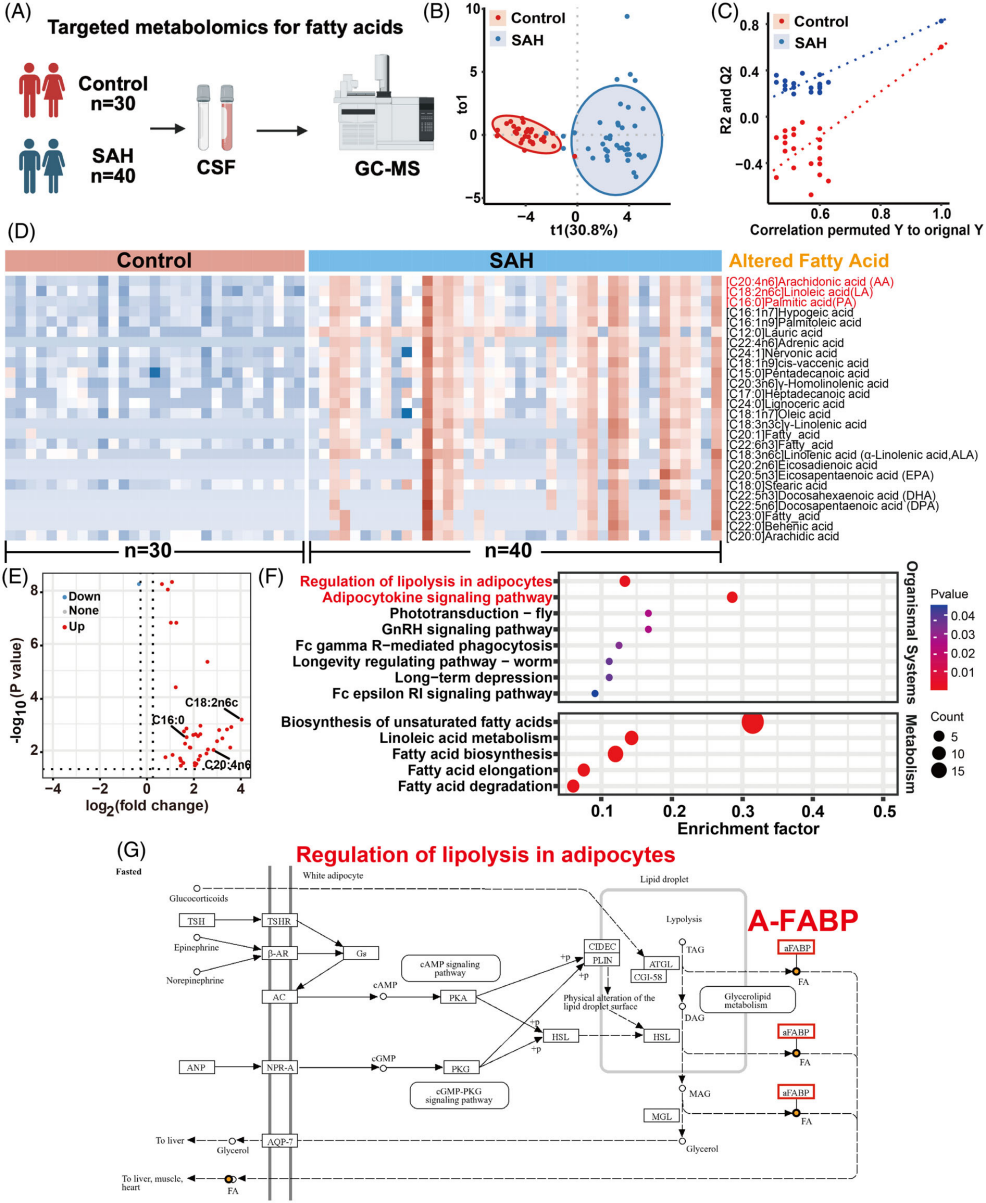
Fatty acid metabolism is significantly disrupted in cerebrospinal fluid (CSF) of SAH patients, with fatty acid‐binding protein (A‐FABP) enrichment. (A) Schematic workflow of targeted fatty acid metabolomics analysis based on human CSF samples (subarachnoid haemorrhage [SAH] group, *n* = 40; control group, *n* = 30). (B and C) The orthogonal partial least squares discrimination analysis (OPLS‐DA) reveals clear separation between the SAH (blue) and control (red) groups. (D) Heatmap of the differentially expressed fatty acid metabolites between the two groups. Rows represent individual fatty acids; colour intensity reflects relative abundance (red: high; blue: low). (E) The volcano plot presents significantly altered fatty acids, including C20:4n6 (arachidonic acid [AA]), C18:2n6c (linoleic acid [LA]) and C16:0 (palmitic acid [PA]). (F) Kyoto Encyclopaedia of Genes and Genomes (https://www.kegg.jp/kegg/pathway.html) (KEGG) pathway enrichment analysis of dysregulated fatty acid metabolism. (G) The regulation of lipolysis in adipocytes in KEGG pathway database (map04923), with A‐FABP highlighted in red. *Source*: Schematic workflow created with BioRender.com.

Targeted fatty acid metabolomics analysis was conducted on 70 CSF samples (including 40 SAH patients and 30 matched controls, Figure 1A). The baseline characteristics are in Table S1. OPLS‐DA (orthogonal partial least squares discriminant analysis) demonstrated high technical reproducibility and revealed a clear separation of fatty acid metabolic profiles in CSF between SAH patients and controls (Figure 1B,C). The heatmap and volcano plot showed that fatty acid metabolism was significantly disrupted in SAH patients compared to controls (Figure 1D,E). Metabolomic profiling identified three core dysregulated fatty acids, including C20:4n6 (AA), C18:2n6c (linoleic acid [LA]) and C16:0 (PA). These fatty acids were strongly linked to the synthesis of inflammatory cytokines, as previously reported.^7^

To further determine the fatty acid metabolic processes occurring in SAH patients, we annotated fatty acid metabolites using the KEGG pathway database. Enrichment analysis revealed two significantly dysregulated pathways: adipocytokine signalling pathway and regulation of lipolysis in adipocytes (Figure 1F). Notably, adipocyte A‐FABP emerged as the sole fatty acid‐associated protein within the pathways (Figure 1G). Crucially, A‐FABP is an intrinsic member of the adipocytokine family. Collectively, our data indicate that fatty acid metabolism is significantly disrupted in CSF of patients with SAH, associated with the enrichment of A‐FABP.

### 3.2 Elevated levels of A‐FABP in CSF predict SAH severity and poor outcomes

To evaluate the clinical value of A‐FABP, we expanded our cohort to include additional SAH patients with complete clinical follow‐up data (total *n* = 48), while maintaining the original 40 patients for targeted fatty acid metabolomics analysis to ensure consistency.

**FIGURE 2:**
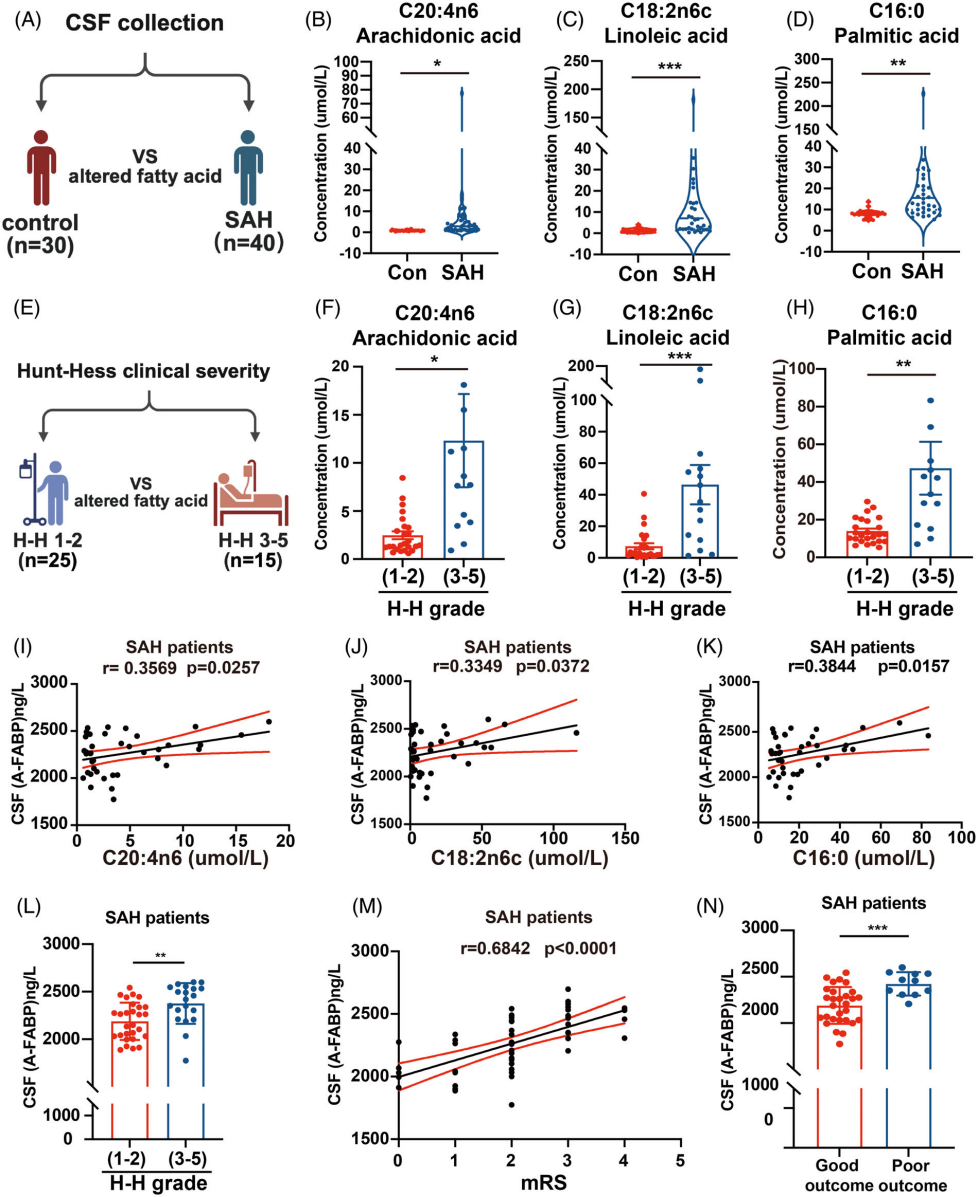
Elevated levels of fatty acid‐binding protein (A‐FABP) in cerebrospinal fluid (CSF) predict subarachnoid haemorrhage (SAH) severity and poor outcomes. (A) Schematic of cohort grouping (SAH: *n* = 40; control: *n* = 30). (B–D) Violin plots present levels of significantly altered fatty acids in CSF: (B) arachidonic acid (AA, C20:4n6), (C) linoleic acid (LA, C18:2n6c) and (D) palmitic acid (PA, C16:0). (E) Schematic of cohort grouping by SAH severity: (Hunt and Hess [H–H] group 1–2 level, *n* = 25; H–H group 3–5 level, *n* = 15). (F–H) Levels of (F) AA, (G) LA and (H) PA between severity low‐grade (H–H 1–2) and high‐grade (H–H 3–5) subgroups. (I–K) Correlation between significantly altered fatty acids and A‐FABP levels in CSF of SAH patients [(I) AA, (J) LA and (K) PA, *n* = 40]. (L) CSF levels of A‐FABP between severity low‐grade (H–H 1–2) and high‐grade (H–H 3–5) subgroups (*n* = 48). (M and N) The relationship between CSF levels of A‐FABP and functional outcomes (modified Rankin Scale [mRS]) in SAH patients (*n* = 48). Data are presented as means ± SD. *p < .05, **p < .01, ***p < .001. *Source*: Schematic diagrams created with BioRender.com.

We first quantified AA, LA and PA in CSF of SAH patients and controls (Figure 2A). Three fatty acids exhibited significant elevations in SAH cohort (Figure 2B–D). To further investigate metabolic‐clinical correlations, the SAH cohort was stratified into two subgroups based on H–H grading: low‐grade (Grades I–II, *n* = 25) and high‐grade (Grades III–V, *n* = 15) (Figure 2E). Analysis revealed significant elevations in AA (Figure 2F), LA (Figure 2G) and PA (Figure 2H) levels in the high‐grade clinical severity cohort compared to low‐grade counterparts. We further sought to determine whether there is a link between elevated fatty acids and A‐FABP in CSF of SAH patients. Pearson's analysis showed a positive correlation between A‐FABP and AA (*r* = .3569, 95% CI = .0467–.6044, *p* = .0257), LA (*r* = .3349, 95% CI = .0217–.5883, *p* = .0372) and PA (*r* = .3844, 95% CI = .0785–.6242, *p* = .0157) (Figure 2 I–K).

To evaluate the clinical potential of A‐FABP, we assessed severity using H–H grade and analysed functional prognosis via the mRS in the SAH cohort (*n* = 48). Our results indicated that A‐FABP levels in CSF correlated with severity (Figure 2L) and worse outcomes (Figure 2M,N). Overall, these findings highlight A‐FABP's potential as a clinical biomarker.

### 3.3 Cerebral A‐FABP is increased in mice and predominantly colocalizes with microglia

**FIGURE 3:**
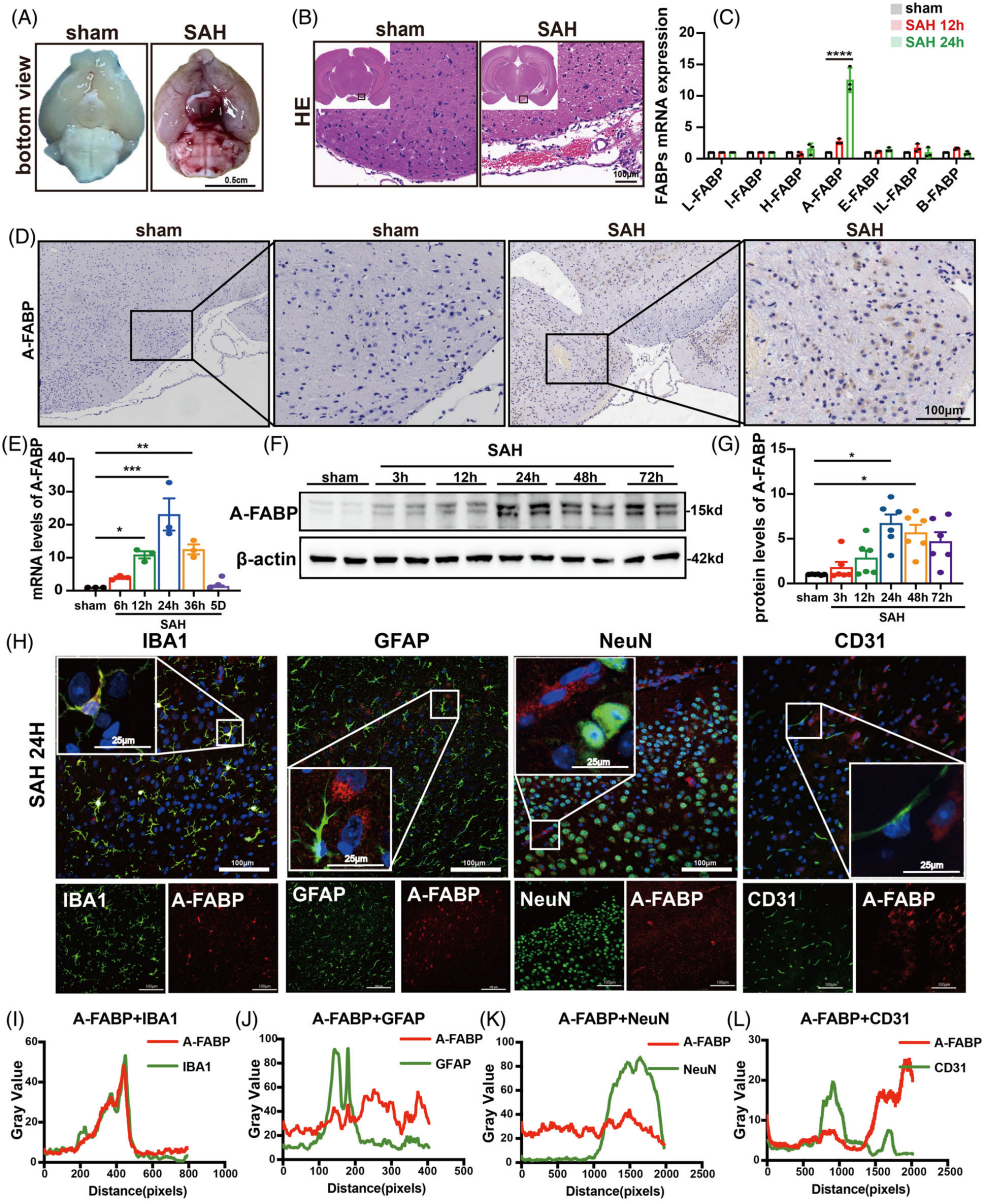
Cerebral fatty acid‐binding protein (A‐FABP) is increased in mice and predominantly colocalizes with microglia. (A and B) Representative photographs from the bottom of mice brains and H&E staining from sham and subarachnoid haemorrhage (SAH) groups. (C) Quantification of FABPs family mRNA level in the mice cortex 24 h after SAH (*n* = 3). (D) Representative images of immunohistochemical staining of A‐FABP (scale bar = 100 µm, *n* = 6). (E) Quantification of A‐FABP mRNA level in the mice cortex after SAH (*n* = 6). (F and G) Western blot images and quantitative analysis of A‐FABP expressions in the mice cortex at 3, 12, 24, 48 and 72 h post‐SAH (*n* = 6). (H–L) Representative microphotographs and quantitative analyses of immunofluorescence co‐staining for A‐FABP (red) with microglia (IBA1, green), astrocyte cells (GFAP, green), neuron cells (NeuN, green) and endothelial cells (CD31, green). Scale bar = 100 µm, *n* = 6. Data are presented as means ± SD. *p < .05, **p < .01, ***p < .001.

To validate the expression and localization of A‐FABP after SAH, we established SAH mice model using endovascular puncture method. Representative images along with H&E staining from both sham and SAH groups are shown in Figure 3A,B. Mortality and excluded mice details are provided in Table S4. The corresponding SAH grading scores are presented in Figure S2. Real‐time polymerase chain reaction (RT‐qPCR) was conducted to evaluate all members of the FABP family. Notably, among the FABPs, A‐FABP exhibited the most significant upregulation in the SAH brain (Figure 3C). Immunohistochemical staining indicated that A‐FABP was expressed around the red blood cells in the subarachnoid space (Figure 3D). Following SAH, A‐FABP mRNA levels were elevated. This upregulation was transient, with expression declining to baseline by Day 5 (Figure 3E). The protein level of A‐FABP started at 3 h and then declined over 72 h (Figure 3F,G). Additionally, immunofluorescence co‐staining analysis was performed to examine the localization of A‐FABP with neuronal cells (NeuN), astrocytes (GFAP), endothelial cells (CD31) and microglia (IBA1), indicating that the resident microglia were the major cellular sources of cerebral A‐FABP following SAH (Figure 3H–L). Together, these findings suggest that the expression of A‐FABP was higher in the SAH mice and predominantly expressed in microglia.

### 3.4 Genetic ablation of A‐FABP ameliorates early brain injury in SAH mice

**FIGURE 4:**
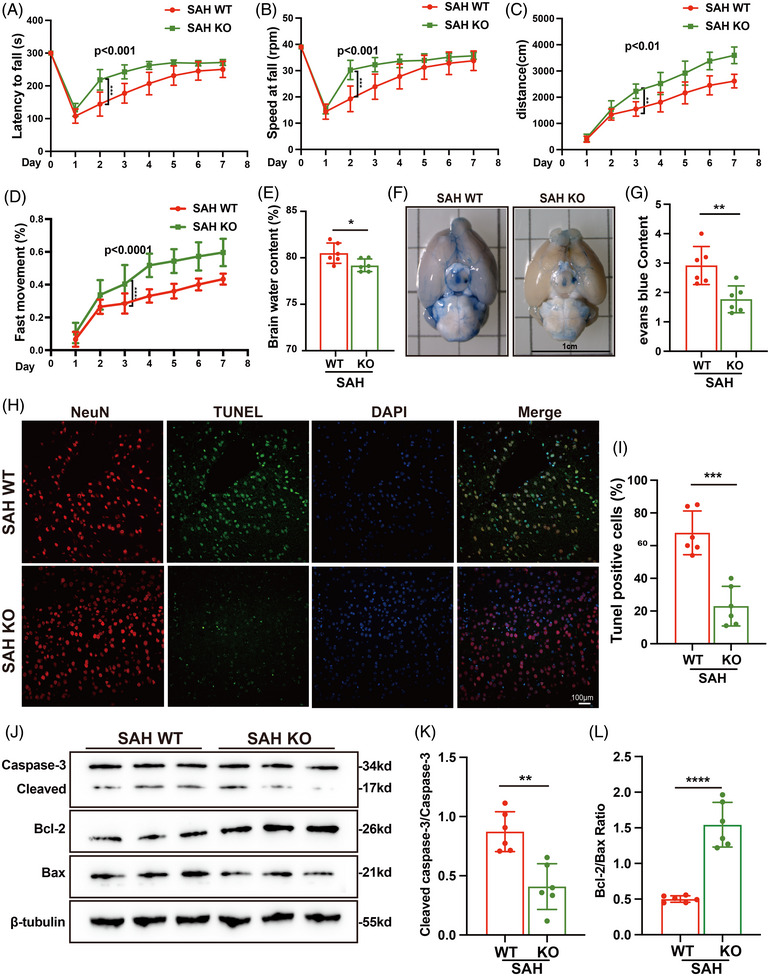
Genetic ablation of fatty acid‐binding protein (A‐FABP) ameliorates brain injury in mice with subarachnoid haemorrhage (SAH). (A and B) Rotarod test: (A) latency to fall on the accelerating rotarod, (B) speed at fall on the accelerating rotarod (*n* = 8). (C and D) Open field test: (C) distance travelled and (D) fast movement proportion (*n* = 8). (E) Percentage of brain water content (*n* = 6). (F and G) Representative photographs and quantitative analysis of mice brain stained with Evans Blue (*n* = 6). (H and I) Representative images and quantitative analysis of TUNEL staining with co‐staining with NeuN (red) and TUNEL‐positive neurons (green, scale bar = 100 µm, *n* = 6). (J and L) Representative western blot images and quantitative analyses of (K) cleaved Caspase‐3/Caspase‐3 and (L) Bcl‐2/Bax (*n* = 6). Data are presented as means ± SD. *p < .05, **p < .01, ***p < .001, ****p < .0001.

To determine the role of A‐FABP in the pathology of SAH, we performed a succession of experiments using WT and A‐FABP KO mice. Identification of genotypes is shown in Figure S3A–C. Neurobehavioural assessments, including the open field and rotarod tests, revealed that neurological deficits after SAH were improved significantly in A‐FABP KO mice when compared with the WT group (Figure 4A–D). Following SAH, A‐FABP KO mice had less brain oedema than WT group (Figure 4E). BBB permeability results demonstrated that KO mice exhibited markedly reduced Evans blue leakage and a more intact BBB (Figure 4F,G). Furthermore, TUNEL staining and analysis of apoptosis‐related protein expression revealed that neuronal apoptosis in the KO mice was attenuated significantly (Figure 4H–L). These data suggest that A‐FABP contributes, at least in part through microglia, to brain injury and thereby to poor functional outcomes after SAH.

### 3.5 Pharmacological inhibition of A‐FABP attenuates brain injury in SAH mice

To determine whether pharmacological inhibition of A‐FABP attenuates brain injury after SAH, mice were subjected to the SAH model and then randomly assigned to receive the selective A‐FABP inhibitor BMS309403 (BMS) or vehicle (Veh) (Figure S4A). Consistent with previous results, BMS significantly improves neurological functions after SAH (Figure S4B–F). BMS also reduced Evans blue leakage (Figure S4G,H), cerebral oedema (Figure S4I) and neuronal apoptosis (Figure S4J–N), suggesting that pharmacological inhibition of A‐FABP possesses a protective effect on brain injury of SAH.

### 3.6 A‐FABP exacerbates microglia‐mediated neuroinflammation in mice with SAH

Previous studies have demonstrated that inhibiting A‐FABP significantly reduces inflammation and exerts therapeutic effects in various diseases.^9^, ^18^ Extending these findings to brain, our colocalization assay showed that microglia became the major source of A‐FABP following SAH (Figure 3H–L), leading us to hypothesize that A‐FABP may promote microglial neuroinflammatory, thereby exacerbating EBI.

**FIGURE 5:**
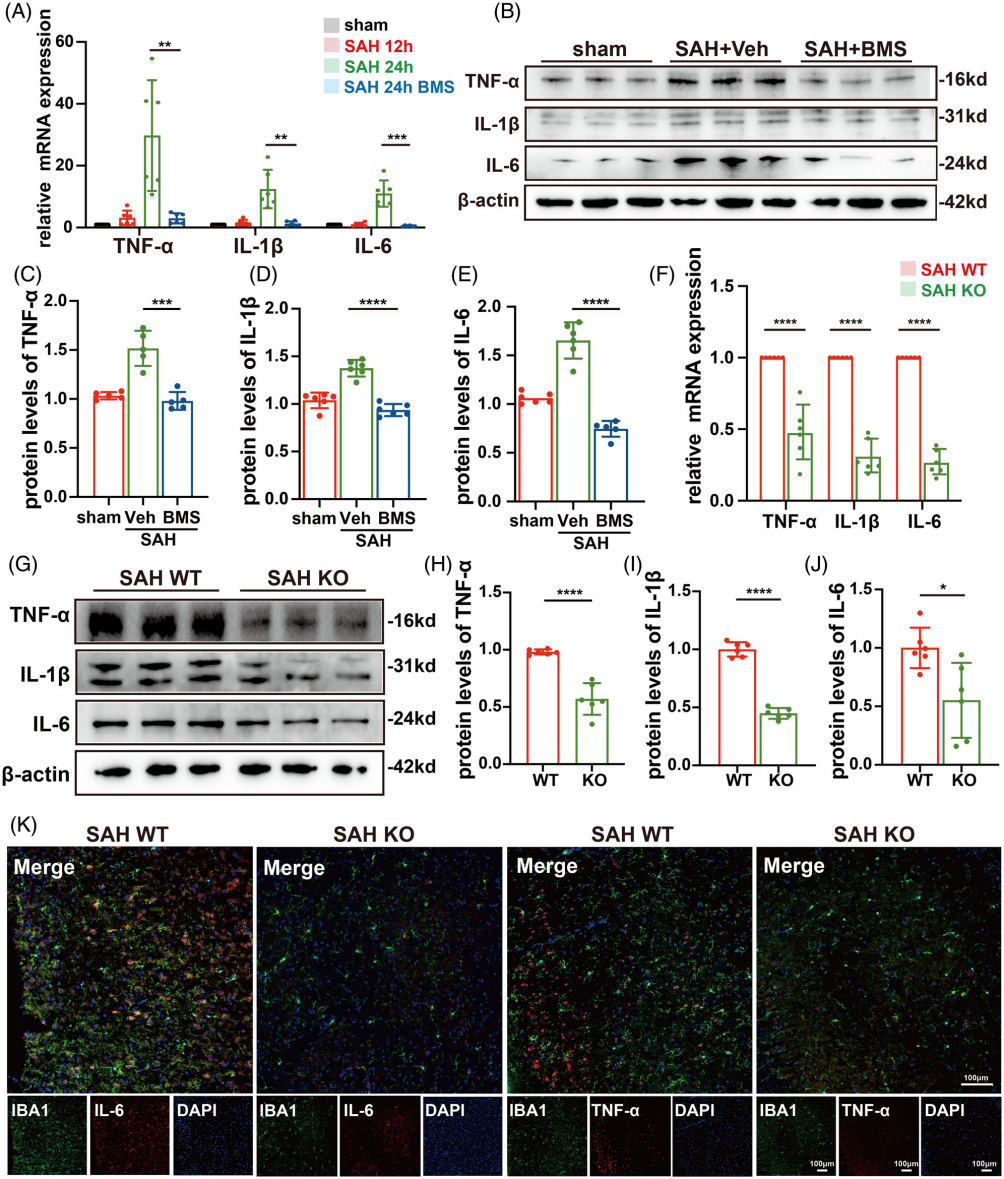
Fatty acid‐binding protein (A‐FABP) exacerbates microglia‐mediated neuroinflammation in mice with subarachnoid haemorrhage (SAH). (A) The mRNA levels of pro‐inflammatory cytokines TNF‐α, IL‐1β and IL‐6 are measured in different groups (*n* = 6). (B–E) Representative western blot images and quantitative analyses of (C) TNF‐α, (D) IL‐1β and (E) IL‐6 in different groups (*n* = 6). (F) The mRNA levels of pro‐inflammatory cytokines TNF‐α, IL‐1β and IL‐6 in WT and knockout (KO) mice after SAH (*n* = 6). (G–J) Representative western blot images and quantitative analyses of pro‐inflammatory cytokines (H) TNF‐α, (I) IL‐1β and (J) IL‐6 in WT and KO mice after SAH (*n* = 6). (K) Representative microphotographs of immunofluorescence co‐staining for pro‐inflammatory cytokines (red) with microglia (IBA1, green) in WT and KO mice after SAH (scale bar = 100 µm, *n* = 6). Data are presented as means ± SD. *p < .05, **p < .01, ***p < .001.

Consistent with the hypothesis, our results demonstrated that BMS administration significantly reduced the levels of inflammatory cytokines, including TNF‐α, IL‐1β and IL‐6 (Figure 5A–E). To validate these pharmacological observations, we further evaluated the expression of inflammatory cytokine in KO mice. Similarly, RT‐qPCR and western blot analyses revealed a decrease in SAH‐induced neuroinflammation in KO mice compared with WT mice (Figure 5F–J). Additionally, double immunofluorescence analysis revealed decreased co‐expression of IL‐6 and TNF‐α with microglial marker IBA1 in A‐FABP‐deficient mice (Figure 5K).

Collectively, these findings indicate that A‐FABP promotes neuroinflammation following SAH and represents a potential therapeutic target for mitigating microglia inflammation following SAH.

### 3.7 A‐FABP, upon fatty acid binding, triggers microglia‐mediated neuroinflammation

**FIGURE 6:**
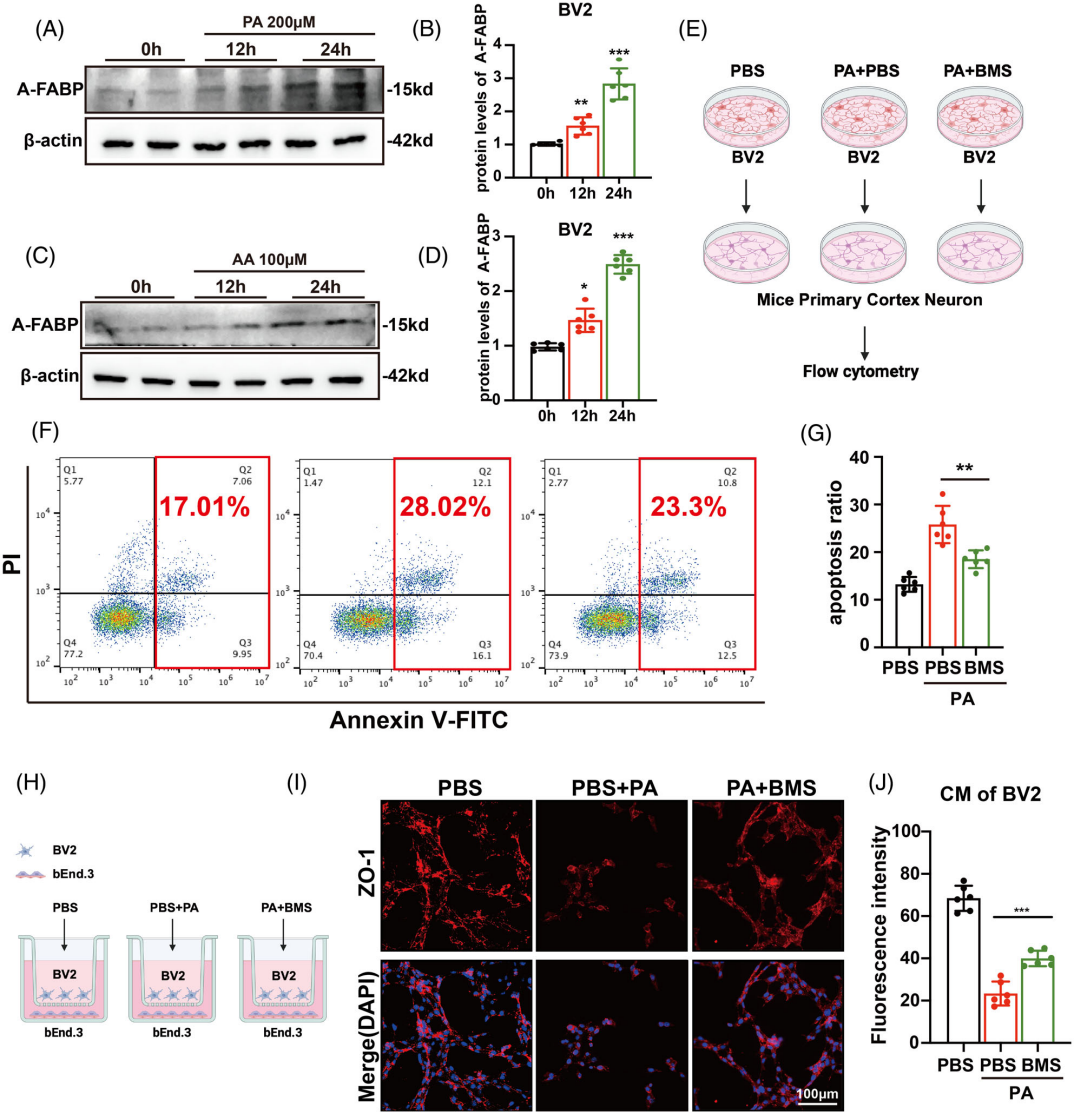
Inhibition of fatty acid‐binding protein (A‐FABP) in microglia attenuates neuronal apoptosis and blood–brain barrier disruption. (A and B) Representative western blot image and quantitative analysis of A‐FABP expression at 0, 12 and 24 h post‐PA (200 µM) treatment (*n* = 6). (C and D) Representative western blot image and quantitative analysis of A‐FABP expression at 0, 12 and 24 h post‐AA (100 µM) treatment (*n* = 6). (E) Schematic diagram of microglial culture supernatant transfer model (PA, 200 µM; BMS, 20 µM). (F and G) Representative flow cytometry images of primary neuron cells apoptosis in PA‐induced microglial culture supernatant transfer model (*n* = 6). (H) Schematic representation of bEnd.3 endothelial cell co‐culture system with BV2 microglial conditioned medium (CM) treated with PA (200 µM), with or without BMS (20 µM). (I and J) Representative microphotographs of immunofluorescence and quantitative analysis in bEnd.3 cells co‐cultured with the CM of BV2 cells (scale bar = 100 µm, *n* = 6). Data are presented as means ± SD. *p < .05, **p < .01, ****p < .0001.

We first assessed A‐FABP expression in BV2 cells treated with PA or AA. Western blotting showed that both PA and AA could upregulate the levels of A‐FABP in BV2 cells (Figure 6A–D). Based on these findings, we next investigated whether fatty acids mediate microglial inflammation via A‐FABP (Figure S5A). Both fatty acids promoted the expression of pro‐inflammatory cytokines. Conversely, inhibition of A‐FABP with BMS significantly attenuated pro‐inflammatory effects induced by fatty acids (Figure S5B–I). Given that both PA and AA induced inflammation in BV2, we exclusively utilized PA in subsequent experiments.

### 3.8 Inhibition of A‐FABP in microglia attenuates neuronal apoptosis and blood–brain barrier disruption

To investigate whether A‐FABP promotes neuronal apoptosis by mediating neuroinflammation, we established a BV2 microglial conditioned medium transfer model. Treated BV2 conditioned medium was transferred to primary mouse neuronal cultures (schematic diagram in Figure 6E). We first identified the primary cortical neurons by immunofluorescence staining (Figure S6). Flow cytometric analysis revealed that conditioned medium from PA‐stimulated BV2 cells significantly increased the proportion of apoptotic neurons. This pro‐apoptotic effect was effectively reversed by BMS treatment (Figure 6F,G).

To further clarify that microglial A‐FABP (not vascular endothelial cells) promotes BBB disruption via neuroinflammation, we established a co‐culture model of microglia and cerebral vascular endothelial bEnd.3 cells (Figure 6H). PA‐stimulated BV2 microglial cells decreased the tight junctions of bEnd.3 cells. Notably, inhibiting A‐FABP mitigated the detrimental effects of PA on tight junctions (Figure 6I,J). Additionally, when bEnd.3 cells were directly exposed to PA, a reduction in tight junction was also observed (Figure S7A). However, inhibiting A‐FABP solely in bEnd.3 cells failed to exert a protective effect on tight junction integrity (Figure S7B–E). These results indicate that A‐FABP promotes neuronal apoptosis and BBB disruption through the regulation of microglia‐mediated neuroinflammation.

### 3.9 A‐FABP suppression promotes fatty acid β‐oxidation reprogramming in microglia

**FIGURE 7:**
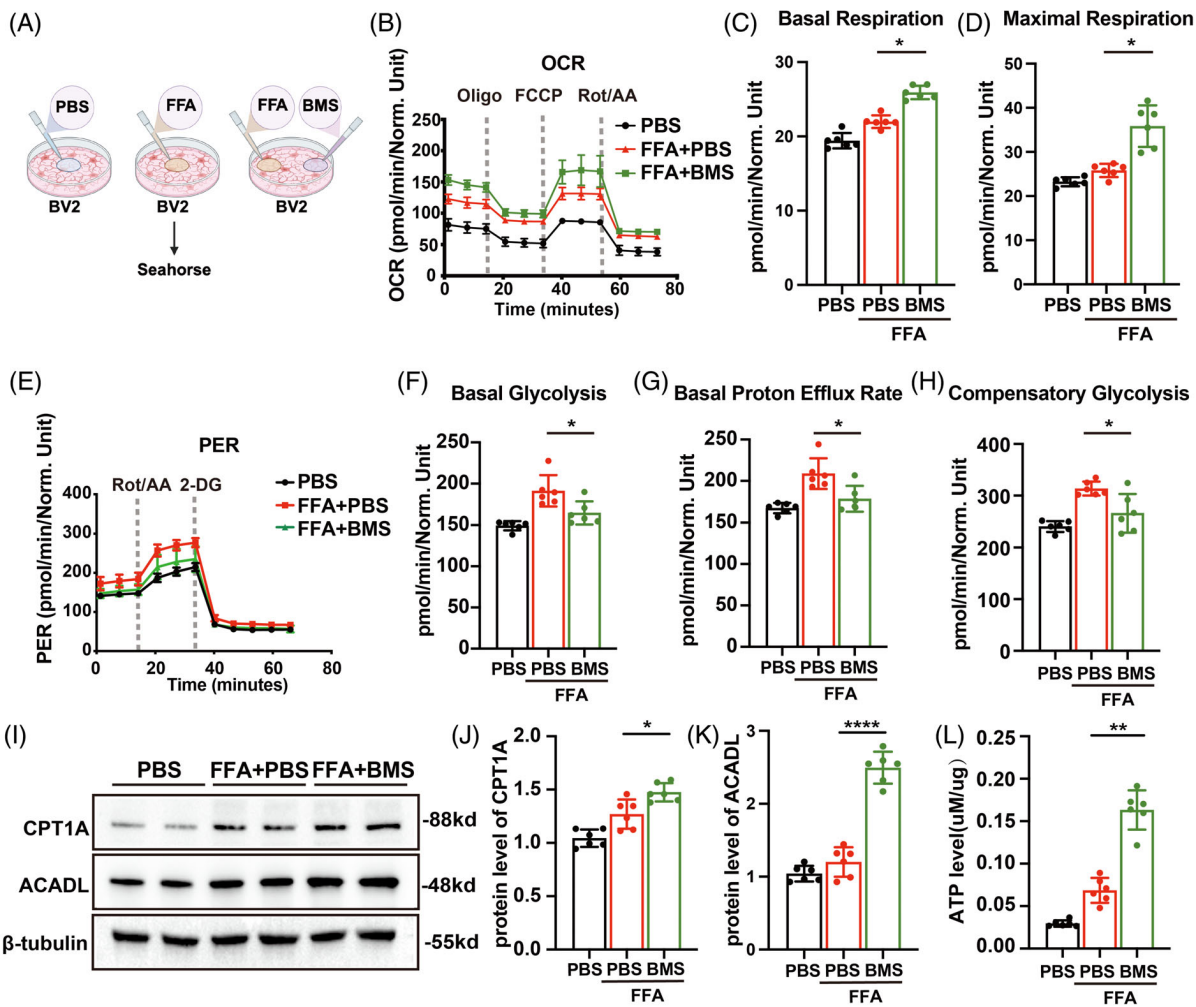
Fatty acid‐binding protein (A‐FABP) suppression promotes fatty acid β‐oxidation reprogramming in microglia. (A) Schematic illustration of experimental design. Control group: PBS treatment. FFA group: 200 µM palmitic acid (PA). BMS group: Co‐treatment with PA (200 µM) and BMS (20 µM). (B–D) Oxygen consumption rate (OCR) was determined in BV2 cells treated with PA, with or without BMS (*n* = 6). Quantitative analyses of (C) basal respiration and (D) maximal respiration. (E–H) Glycolytic proton efflux rate (GlycoPER) was determined in BV2 cells treated with PA, with or without BMS (*n* = 6). Quantitative analyses of (F) basal glycolysis, (G) basal proton efflux rate and (H) compensatory glycolysis. (I–K) Representative western blot images and quantitative analyses of (J) CPT1A and (K) ACADL in different groups (*n* = 6). (L) ATP content assay in different groups (*n* = 6). Data are presented as means ± SD. *p < .05, ****p < .0001. glycoPER, glycolytic proton efflux rate. *Source*: Schematic diagram created with BioRender.com.

Subsequently, we sought to determine whether A‐FABP modulates metabolic reprogramming exacerbating cerebral injury (Figure 7A). Seahorse XF analysis showed that PA stimulation did not alter cellular OCR, whereas BMS treatment significantly increased OCR levels (Figure 7B–D). Glycolysis assays indicated that FA stimulation increased microglial glycolytic proton efflux rate (GlycoPER), but BMS treatment counteracted this effect (Figure 7E–H). Western blot analysis further confirmed that BMS treatment increased fatty acid β‐oxidation (CPT1A and ACADL in Figure 7I–K). ATP level detection confirmed that BMS treatment enhanced cellular energy supply (Figure 7L).

Our findings indicate that fatty acids promote neuroinflammation through binding with A‐FABP. Competitive occupation of the A‐FABP ligand‐binding domain by BMS enhances fatty acid β‐oxidation, thereby improving energy supplement.

### 3.10 A‐FABP mediates neuroinflammation via the JAK2/STAT3 signalling pathway

**FIGURE 8:**
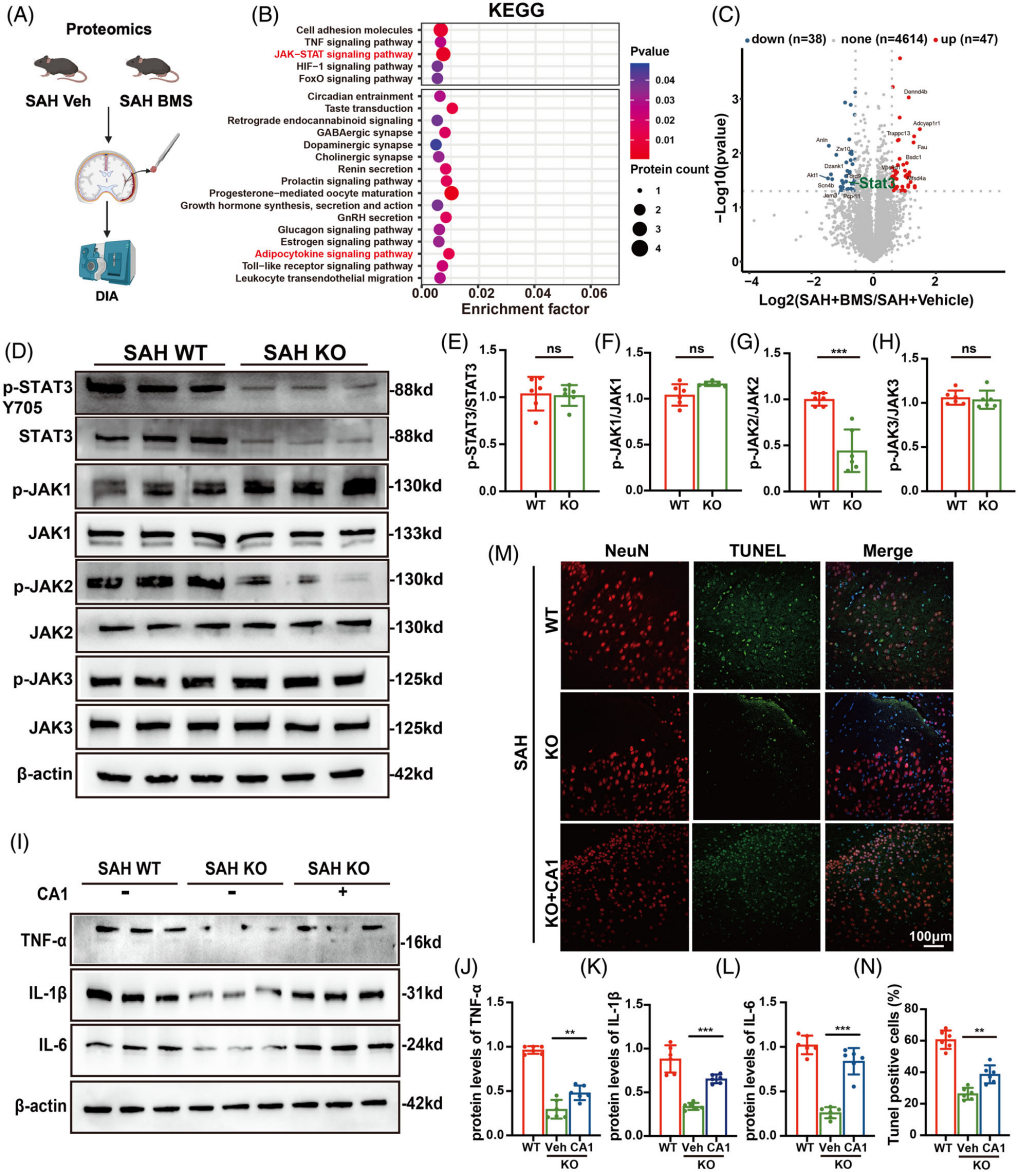
Fatty acid‐binding protein (A‐FABP) mediates neuroinflammation via the JAK2/STAT3 signalling pathway. (A) Workflow diagram. Proteomic analysis of brain tissue around the subarachnoid space from the subarachnoid haemorrhage (SAH)‐treated Veh group and the SAH‐treated BMS group (*n* = 4). (B) KEGG enrichment highlights the JAK‐STAT signalling pathway. (C) Volcano plot demonstrates the results of 85 differentially expressed proteins. STAT3 is labelled in green. (D–H) Representative western blot images and quantitative analyses of (E) p‐STAT3/STAT3, (F) p‐JAK1/JAK1, (G) p‐JAK2/JAK2 and (H) p‐JAK3/JAK3 in WT and knockout (KO) mice after SAH. (I–L) Representative western blot images and quantitative analyses of (J) TNF‐α, (K) IL‐1β and (L) IL‐6 in different groups. (M and N) Representative images and quantitative analysis of TUNEL staining with co‐staining with NeuN (red) and TUNEL‐positive neurons (green, scale bar = 100 µm, *n* = 6). Data are presented as means ± SD. *p < .05, **p < .01, ***p < .001. C‐A1, coumermycin A1; ns, no significance. *Source*: Schematic diagram created with BioRender.com.

To delineate the downstream mechanisms by which A‐FABP regulates neuroinflammation post‐SAH, we performed proteomics on subarachnoid brain tissues from SAH + Veh and SAH + BMS groups (Figure 8A). We identified 85 differentially expressed proteins (47 upregulated, 38 downregulated). KEGG pathway enrichment analysis revealed predominant involvement of the JAK‐STAT signalling pathway, with STAT3 as a key differentially expressed protein in volcano plot analysis (Figure 8B,C).

Western blot analysis of all JAK family members revealed selective alteration of phosphorylated JAK2 (p‐JAK2) in KO mice, suggesting activation of the JAK2/STAT3 pathway following SAH (Figure 8D–H). Total STAT3 protein levels were also reduced, aligning with proteomic findings. Consistently, pharmacological inhibition of A‐FABP with BMS also demonstrated the similar trend (Figure S8A–C). Functional rescue experiments using the JAK2 agonist coumermycin A1 (CA1) in A‐FABP KO mice reversed both neuroinflammation (Figure 8I–L) and neuronal apoptosis mediated by A‐FABP deletion (Figure 8 M,N). Taken together, these findings confirm that A‐FABP promotes neuroinflammation and subsequently induces apoptosis through the activation of the JAK2/STAT3 signalling pathway.

## 4 DISCUSSION

Previous clinical studies have demonstrated a significant association between elevated circulating A‐FABP levels and adverse clinical outcomes in stroke, including poor functional recovery and increased mortality.^12^ However, these findings failed to provide mechanistic insights into how A‐FABP might exacerbate brain injury. In this study, we revealed that A‐FABP plays a deleterious role in SAH, not only its pathogenic function but also the underlying mechanistic pathways (graphical abstract).

In clinical findings, fatty acid metabolism was significantly disrupted in CSF, including AA, LA and PA, concomitant with A‐FABP enrichment. Furthermore, elevated levels of A‐FABP demonstrated predictive value for SAH severity and unfavourable outcomes. Although A‐FABP has been identified as an adverse prognostic in cardiovascular diseases, ischaemic stroke and intracerebral haemorrhage,^19^, ^20^, ^21^, ^22^ this study is the first time to evaluate A‐FABP as a biomarker in the CSF of SAH patients. Unlike previous studies focused on serum biomarkers, our targeted analysis of CSF offers direct access to the central nervous system—overcoming the limitations of peripheral blood for neurological pathology assessment. Consistent with clinical practice, lumbar puncture is a routine approach for the diagnosis and treatment of SAH which facilitates CSF sample collection. Thus, our data support the candidacy of CSF A‐FABP as a biomarker for SAH.

In vivo, our study revealed significant upregulation of A‐FABP in microglia after SAH. Crucially, evidence from both genetic KO and pharmacological blockade of A‐FABP converged to demonstrate its pathogenic role in SAH. To date, nimodipine remains the only FDA‐approved therapy for improving SAH prognosis, underscoring the urgent need for novel therapeutic medications.^23^ Given its crucial role in various diseases, A‐FABP has emerged as a therapeutic target, prompting the development of small molecule inhibitors.^24^, ^25^, ^26^ Among these, BMS309403, a high‐affinity competitive inhibitor targeting the fatty acid‐binding pocket of A‐FABP, represents the most well‐characterized inhibitor. Our data demonstrate that BMS treatment significantly attenuates SAH‐induced neurological deficits, providing robust preclinical evidence for A‐FABP as a druggable target. Furthermore, our findings also provide a compelling reason for developing higher specificity modalities such as monoclonal antibodies (mAbs). Promisingly, existing A‐FABP‐targeting mAbs (e.g., 6H2 in ischaemic) have shown clinical potential.^27^ These findings position A‐FABP‐targeting mAbs as a promising therapeutic approach for SAH, warranting immediate preclinical development.

Mechanistically, we found that A‐FABP, upon binding to fatty acid, triggers microglia‐mediated neuroinflammation by JAK2/STAT3 pathway. This is consistent with established knowledge: Extracellular matrix inflammation is a feature of ruptured intracranial aneurysm,^28^, ^29^ and aneurysm rupture triggers immediate neuroinflammatory cascades that exacerbate neuronal injury. Crucially, fatty acids and pro‐inflammatory cytokines have recently emerged as biomarkers for unruptured aneurysm instability risk assessment.^7^ Although elevated serum free fatty acids have been shown to independently predict stroke outcomes,^30^, ^31^, ^32^ their underlying role in SAH remains poorly characterized. Logically, A‐FABP exhibits enhanced fatty acid‐binding affinity that potentiates inflammatory responses.^33^ The present study showed that A‐FABP inhibition impeded experimental SAH mice brain injury accompanied by alleviated neuroinflammation. Notably, as BMS inhibits the binding of fatty acids to A‐FABP, it is reasonable to conclude that A‐FABP, rather than fatty acids, serves as the primary mediator of damage following SAH.

Metabolic reprogramming is a key driver of inflammatory phenotypic shifts. Previous research showed that when astrocytic mitochondrial β‐oxidation is blocked, long‐chain fatty acids stop being oxidized. They accumulate as lipid droplets, trigger inflammation and drive neurodegeneration.^34^ Extending this to SAH, we propose that injury‐released free fatty acids are bound by A‐FABP in microglia. This may block their entry into the mitochondrial β‐oxidation pathway, simultaneously disrupting energy metabolism and fuelling neuroinflammation. However, these mechanistic links remain to be proven.

Although our findings provide valuable insights, this study possesses certain limitations. First, although immunofluorescence co‐localization provided preliminary evidence for the cellular sources of A‐FABP, the subsequent animal models were limited by insufficient target specificity. Future investigations should utilize a microglia‐specific A‐FABP conditional KO mouse model to confirm our findings.

Additionally, we acknowledge the relatively modest sample size of our CSF cohort, particularly in control subjects, so bias cannot be ruled out. We recognize that future studies with larger, prospectively matched cohorts will be valuable for confirming these findings. Our ongoing multi‐centre collaboration aims to collect such balanced samples to further validate the role of A‐FABP in SAH pathophysiology. Furthermore, measuring A‐FABP in CSF of SAH mice would further strengthen the translational link. However, the extremely limited CSF volume made a systematic analysis impossible. This does not change our main conclusion about A‐FABP's role in the brain.

In summary, our study reveals that SAH patients exhibit CSF fatty acid metabolism disruption accompanied by A‐FABP enrichment. Genetic ablation or pharmacological inhibition of A‐FABP alleviates the consequences of brain injury following SAH. Collectively, our findings suggest that A‐FABP may hold dual promise as a clinical biomarker and a therapeutic target in SAH.

## AUTHOR CONTRIBUTIONS

All authors contributed to the paper, satisfying the ICMJE guidelines for authorship. Xingwu Liu and Shenquan Guo conducted the experiments and drafted the manuscript. Xin Feng, Hao Tian, Boyang Wei, Lei Jin and Wenchao Liu designed the experiments and data analysis. Jingjing Kong collected clinical sample. Xin Zhang, Ran Li and Zhiyuan Zhu performed writing—review and editing. Lingling Shu, Xifeng Li and Chuanzhi Duan performed study supervision and critical revision of the manuscript.

## CONFLICT OF INTEREST STATEMENT

The authors declare no conflicts of interest.

## ETHICS STATEMENT

This study involves human participants and was approved by the institutional review board of Zhujiang Hospital (2023‐KY‐280‐01). The study was conducted in accordance with the Declaration of Helsinki. Consent to Participate declaration: not applicable. The animal study was approved by the Ethics Committee of Zhujiang Hospital of Southern Medical University (Ethics Project Number LAEC‐2023‐002). All experimental procedures involving animals were conducted in accordance with the National Institutes of Health Guide for the Care and Use of Laboratory Animals.

## Supporting information




**Figure S1. Experimental design and animal grouping**. C‐A1, Coumermycin A1; IF, immunofluorescence; IHC, immunohistochemical staining; KO, knockout; QPCR, quantitative real‐time polymerase chain reaction; SAH, subarachnoid haemorrhage; TUNEL, terminal deoxynucleotidyl transferase dUTP nick end labelling; WB, western blot; WT, wild type.

**Figure S2. Mortality and subarachnoid haemorrhage (SAH) grade**. (A and B) SAH grade scores of all SAH groups. Data are presented as means ± SD. ns, no significance.

**Figure S3. Identification of A‐FABP KO mice**. (A) Typical PCR result of genotyping analysis using specific primers to identify WT and KO mice. (B) No expression of A‐FABP was detected in the brain of KO mice after SAH (*n* = 3). (C) Representative IHC staining of A‐FABP in WT and KO mice after SAH (*n* = 3, scale bar = 100 µm).

**Figure S4. Pharmacological inhibition of A‐FABP attenuates brain injury in mice with SAH**. (A) Schematic of A‐FABP inhibition by vehicle (Veh) or BMS309403 (BMS). SAH mice are treated with Veh/BMS 1‐, 12‐h post‐surgery and then daily. (B and C) Rotarod test: (B) latency to fall on the accelerating rotarod, (C) speed at fall on the accelerating rotarod (*n* = 8). (D–F) Open field test: (D) distance travelled, (E) fast movement proportion during the 7 days and (F) representative photographs of movement distance (*n* = 8). (G and H) Representative photographs and quantitative analysis of mice brain stained with Evans Blue (*n* = 6). (I) Percentage of brain water content (*n* = 6). (J and K) Representative images and quantitative analysis of TUNEL staining with co‐staining with NeuN (red) and TUNEL‐positive neurons (green, scale bar = 100 µm, *n* = 6). (L–N) Representative western blot images and quantitative analyses of (M) cleaved Caspase‐3/Caspase‐3 and (N) Bcl‐2/Bax (*n* = 6). Data are presented as means ± SD. *p < .05, **p < .01, ***p < .001.

**Figure S5. A‐FABP, upon fatty acid binding, triggers microglia‐mediated neuroinflammation**. (A) Schematic diagram of BV2 incubation with different FFAs (PA, 200 µM; AA, 100 µM) and with/without BMS (20 µM). (B–E) Representative western blot images and quantitative analyses of (C) TNF‐α, (D) IL‐1β and (E) IL‐6 treated with PA (200 µM) with or without BMS (20 µM, *n* = 6). (F–I) Representative western blot images and quantitative analyses of (G) TNF‐α, (H) IL‐1β and (I) IL‐6 treated with AA (100 µM) with or without BMS (20 µM, *n* = 6). Data are presented as means ± SD. *p < .05, **p < .01, ***p < .001. AA, arachidonic acid; PA, palmitic acid. *Source*: Schematic diagram created with BioRender.com.

**Figure S6. Identification of primary neuron cells**. (A) Representative images of primary mouse neuron cells. Immunofluorescence double staining of neuron marker (NeuN, green) and astrocyte marker (GFAP, red), counterstained by DAPI (blue). Scale bar = 100 µm.

**Figure S7. A‐FABP promotes blood–brain barrier disruption through the regulation of microglia (not vascular endothelial cells)**. (A) Schematic diagram of bEnd.3 in original medium treated with PA (200 µM), with or without BMS (20 µM). (B and C) Representative microphotographs of immunofluorescence and quantitative analysis in bEnd.3 cells with original medium (scale bar = 100 µm, *n* = 6). (D and E) Representative western blot image and quantitative analysis of occludin (*n* = 6). Data are presented as means ± SD. *p < .05, **p < .01, ***p < .001. CM, conditioned medium. *Source*: Schematic diagrams created with BioRender.com.

**Figure S8. Pharmacological inhibition of A‐FABP suppresses the activation of JAK2/STAT3 signalling pathway**. (A–C) Representative western blot images and quantitative analyses of STAT3, p‐STAT3, JAK2 and p‐JAK2 in different groups after SAH (*n* = 6). Data are presented as means ± SD. ****p < .0001, ns, no significance.

Supporting Information

Supporting Information

Supporting Information

Supporting Information

## Data Availability

Data are available upon reasonable request. The data supporting this study's findings are available from the corresponding author upon reasonable request.
