# Supplementary figures and images for: Adipocyte fatty acid‐binding protein as a cerebrospinal fluid–accessible biomarker and druggable target in subarachnoid haemorrhage: Linking fatty acid dysregulation to microglial neuroinflammation
Source: Clin Transl Med. 2026 Jan 30;16(2):e70607. doi: 10.1002/ctm2.70607 (PMC12856223; doi:10.1002/ctm2.70607)

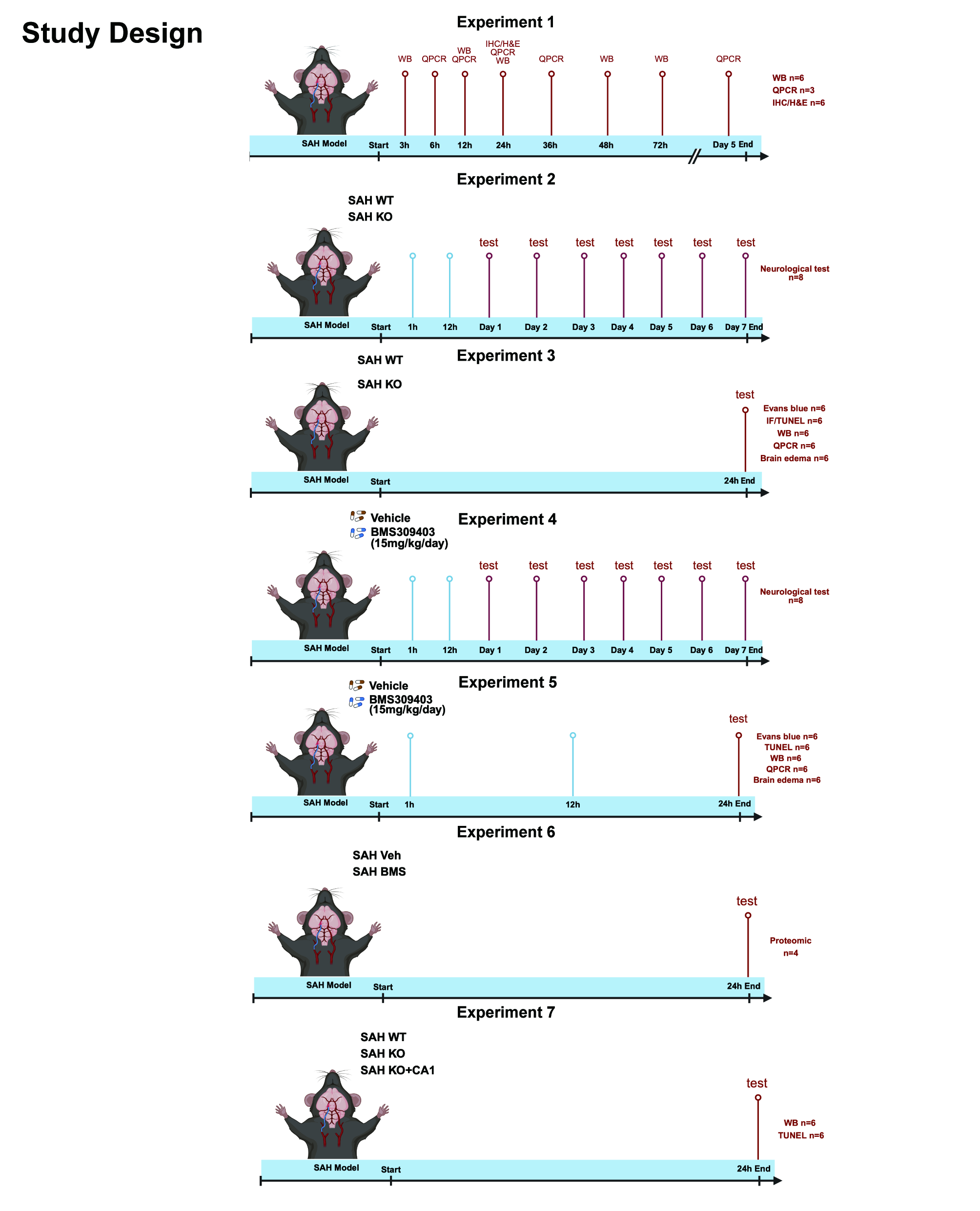

Supplement: Supplementary file 1 — Figure S1. Experimental design and animal grouping. C‐A1, Coumermycin A1; IF, immunofluorescence; IHC, immunohistochemical staining; KO, knockout; QPCR, quantitative real‐time polymerase chain reaction; SAH, subarachnoid haemorrhage; TUNEL, terminal deoxynucleotidyl transferase dUTP nick end labelling; WB, western blot; WT, wild type. [file CTM2-16-e70607-s001.tif]

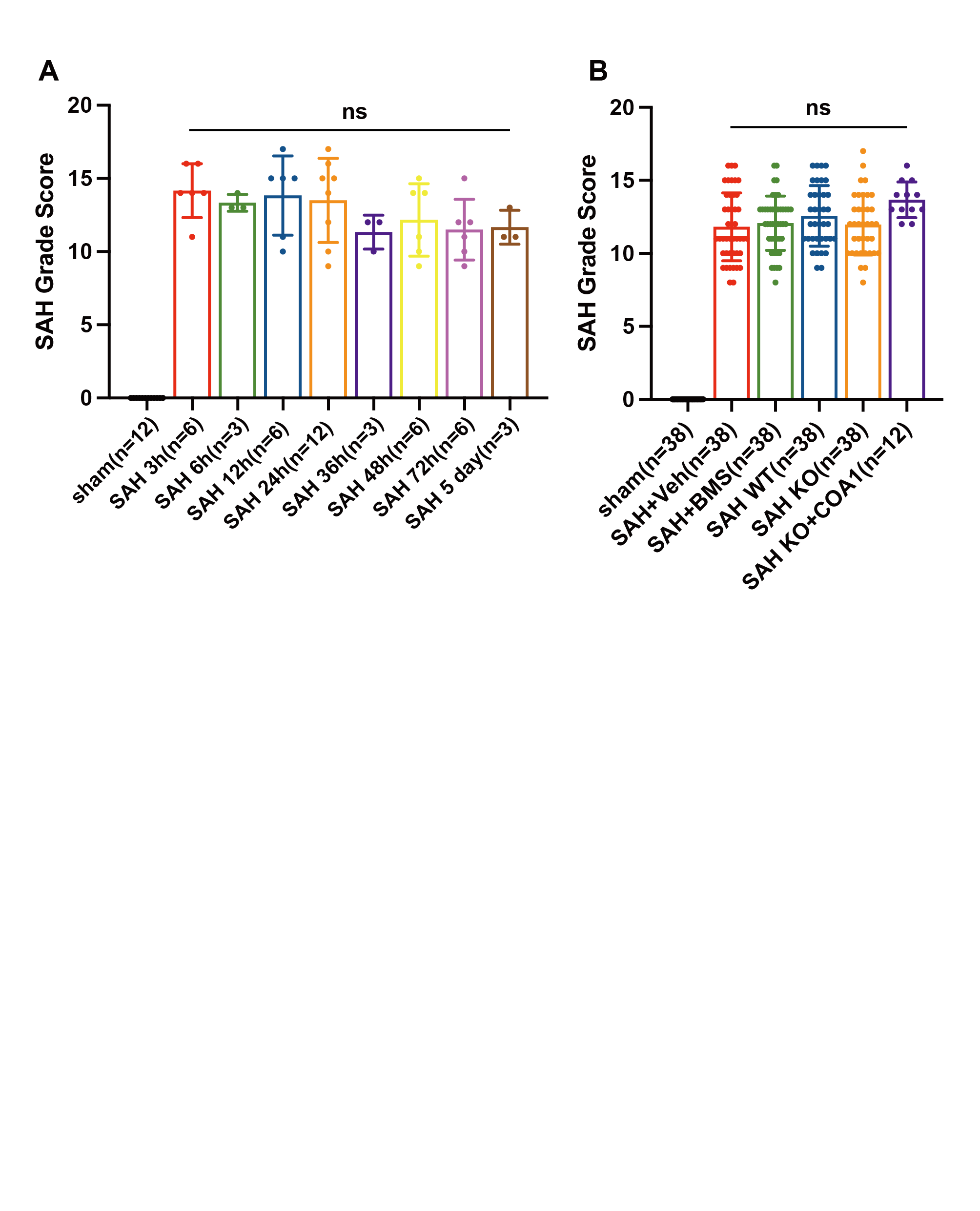

Supplement: Supplementary file 2 — Figure S2. Mortality and subarachnoid haemorrhage (SAH) grade. (A and B) SAH grade scores of all SAH groups. Data are presented as means ± SD. ns, no significance. [file CTM2-16-e70607-s005.tif]

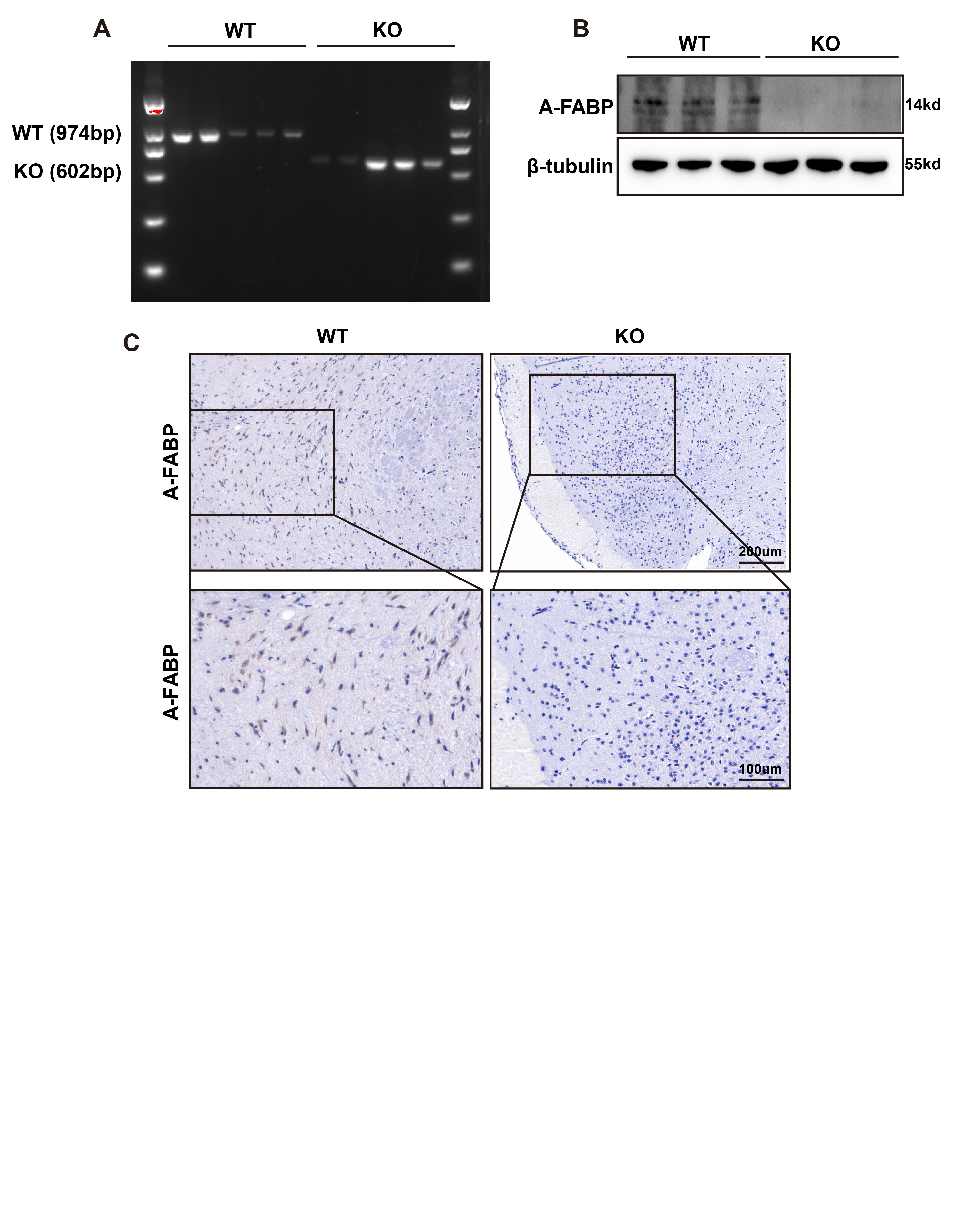

Supplement: Supplementary file 3 — Figure S3. Identification of A‐FABP KO mice. (A) Typical PCR result of genotyping analysis using specific primers to identify WT and KO mice. (B) No expression of A‐FABP was detected in the brain of KO mice after SAH (n = 3). (C) Representative IHC staining of A‐FABP in WT and KO mice after SAH (n = 3, scale bar = 100 µm). [file CTM2-16-e70607-s004.tiff]

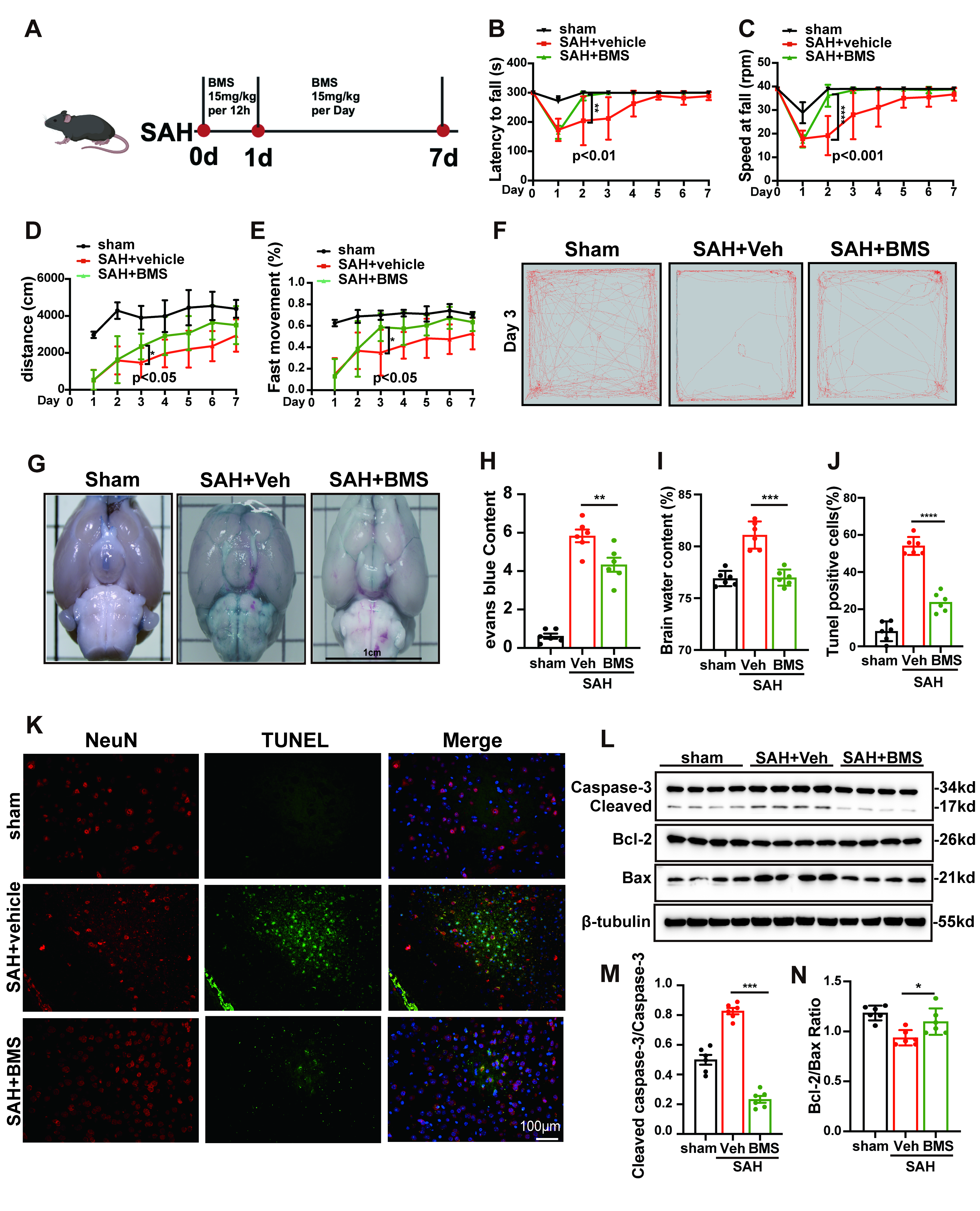

Supplement: Supplementary file 4 — Figure S4. Pharmacological inhibition of A‐FABP attenuates brain injury in mice with SAH. (A) Schematic of A‐FABP inhibition by vehicle (Veh) or BMS309403 (BMS). SAH mice are treated with Veh/BMS 1‐, 12‐h post‐surgery and then daily. (B and C) Rotarod test: (B) latency to fall on the accelerating rotarod, (C) speed at fall on the accelerating rotarod (n = 8). (D–F) Open field test: (D) distance travelled, (E) fast movement proportion during the 7 days and (F) representative photographs of movement distance (n = 8). (G and H) Representative photographs and quantitative analysis of mice brain stained with Evans Blue (n = 6). (I) Percentage of brain water content (n = 6). (J and K) Representative images and quantitative analysis of TUNEL staining with co‐staining with NeuN (red) and TUNEL‐positive neurons (green, scale bar = 100 µm, n = 6). (L–N) Representative western blot images and quantitative analyses of (M) cleaved Caspase‐3/Caspase‐3 and (N) Bcl‐2/Bax (n = 6). Data are presented as means ± SD. *p < .05, **p < .01, ***p < .001. [file CTM2-16-e70607-s011.tif]

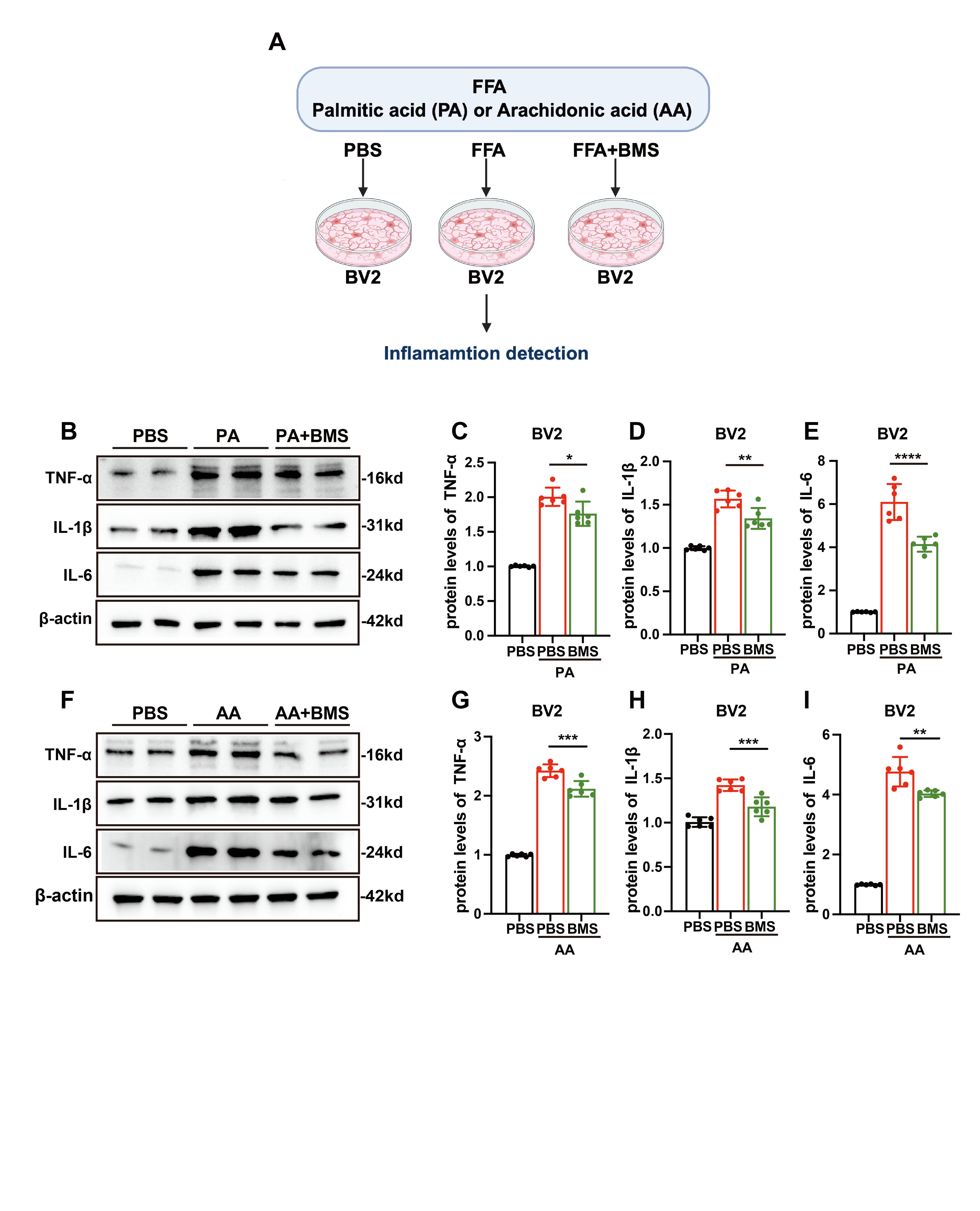

Supplement: Supplementary file 5 — Figure S5. A‐FABP, upon fatty acid binding, triggers microglia‐mediated neuroinflammation. (A) Schematic diagram of BV2 incubation with different FFAs (PA, 200 µM; AA, 100 µM) and with/without BMS (20 µM). (B–E) Representative western blot images and quantitative analyses of (C) TNF‐α, (D) IL‐1β and (E) IL‐6 treated with PA (200 µM) with or without BMS (20 µM, n = 6). (F–I) Representative western blot images and quantitative analyses of (G) TNF‐α, (H) IL‐1β and (I) IL‐6 treated with AA (100 µM) with or without BMS (20 µM, n = 6). Data are presented as means ± SD. *p < .05, **p < .01, ***p < .001. AA, arachidonic acid; PA, palmitic acid. Source: Schematic diagram created with BioRender.com. [file CTM2-16-e70607-s006.tiff]

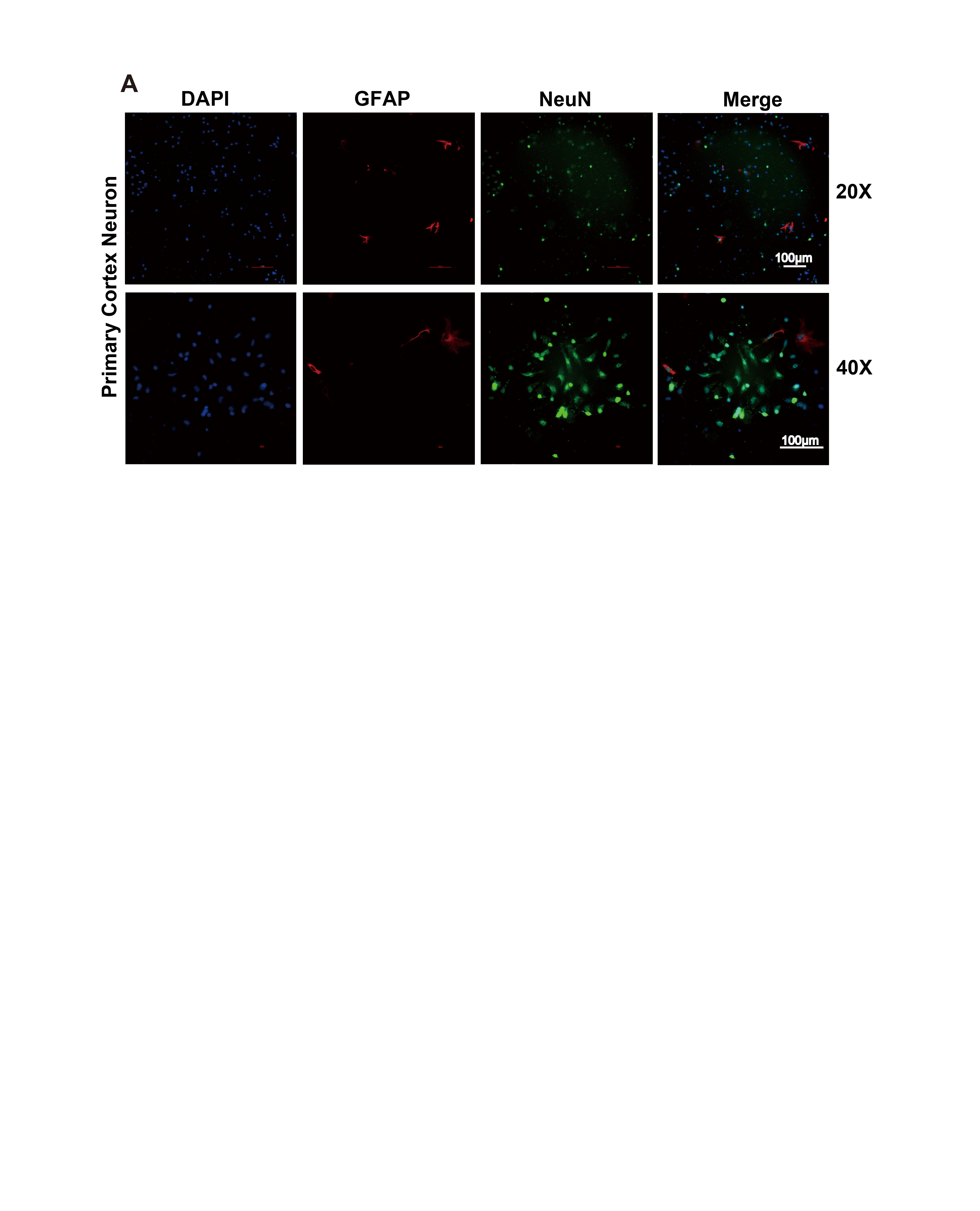

Supplement: Supplementary file 6 — Figure S6. Identification of primary neuron cells. (A) Representative images of primary mouse neuron cells. Immunofluorescence double staining of neuron marker (NeuN, green) and astrocyte marker (GFAP, red), counterstained by DAPI (blue). Scale bar = 100 µm. [file CTM2-16-e70607-s008.tiff]

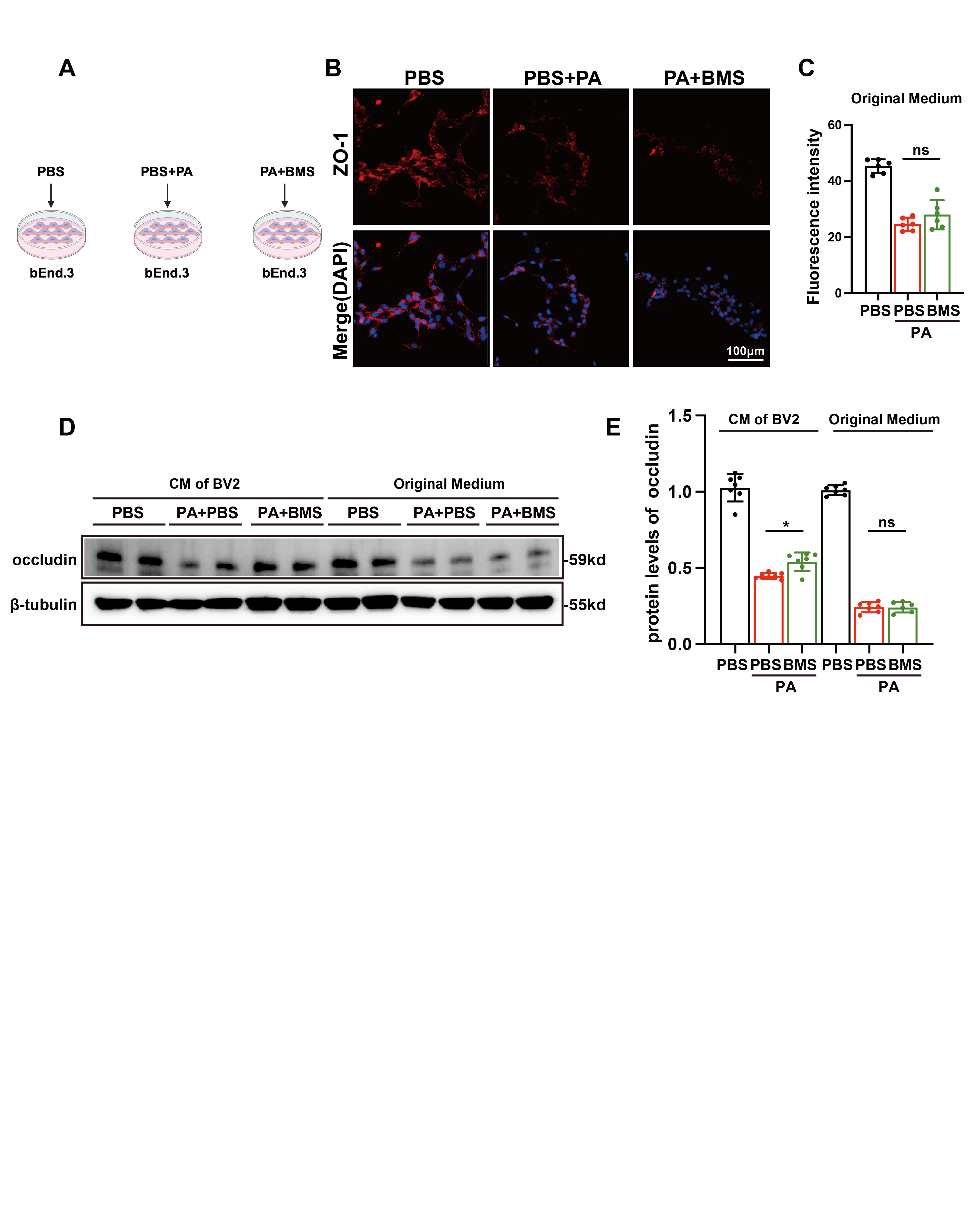

Supplement: Supplementary file 7 — Figure S7. A‐FABP promotes blood–brain barrier disruption through the regulation of microglia (not vascular endothelial cells). (A) Schematic diagram of bEnd.3 in original medium treated with PA (200 µM), with or without BMS (20 µM). (B and C) Representative microphotographs of immunofluorescence and quantitative analysis in bEnd.3 cells with original medium (scale bar = 100 µm, n = 6). (D and E) Representative western blot image and quantitative analysis of occludin (n = 6). Data are presented as means ± SD. *p < .05, **p < .01, ***p < .001. CM, conditioned medium. Source: Schematic diagrams created with BioRender.com. [file CTM2-16-e70607-s007.tiff]

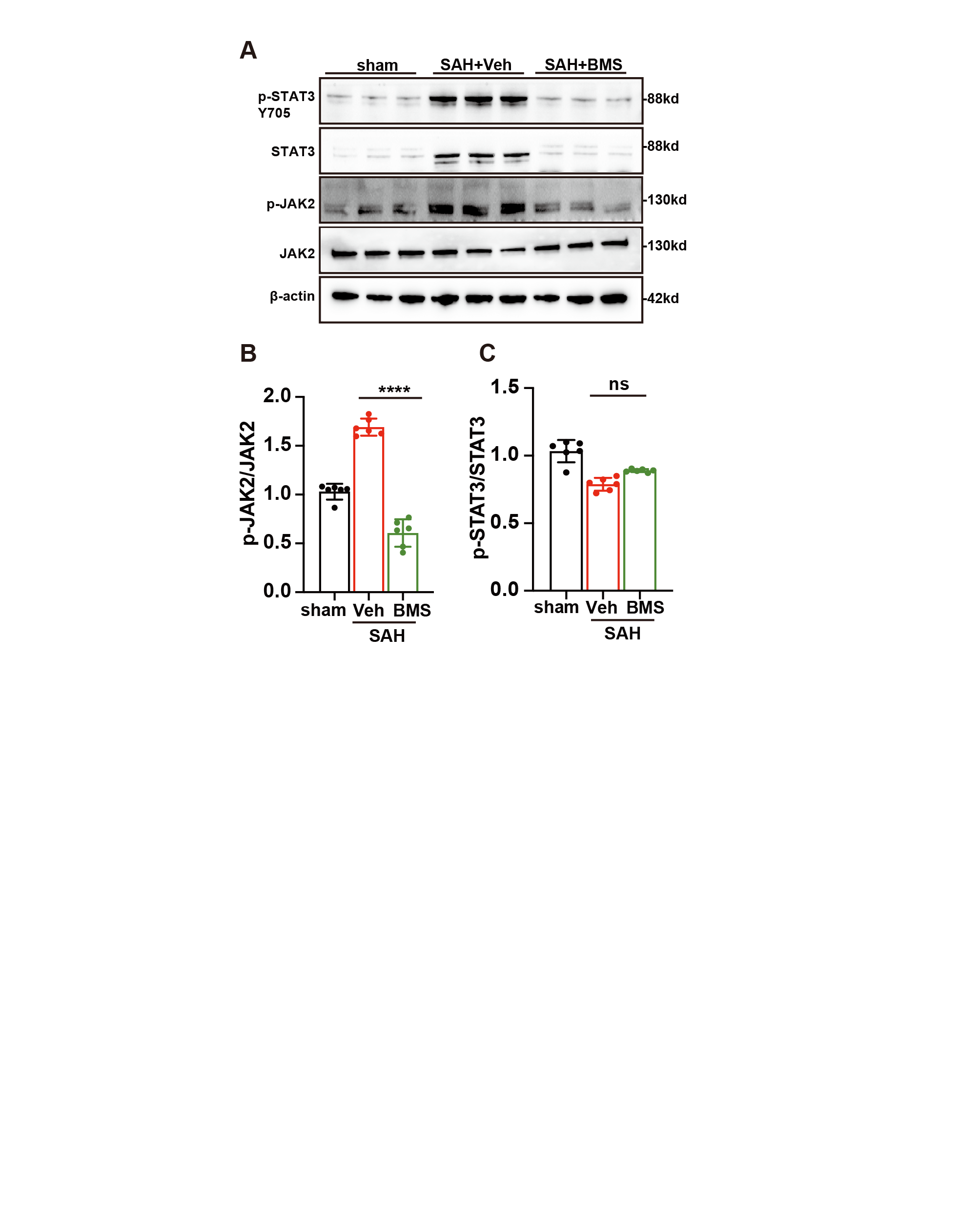

Supplement: Supplementary file 8 — Figure S8. Pharmacological inhibition of A‐FABP suppresses the activation of JAK2/STAT3 signalling pathway. (A–C) Representative western blot images and quantitative analyses of STAT3, p‐STAT3, JAK2 and p‐JAK2 in different groups after SAH (n = 6). Data are presented as means ± SD. ****p < .0001, ns, no significance. [file CTM2-16-e70607-s010.tiff]
