# Supplementary material for: Adipocyte fatty acid‐binding protein as a cerebrospinal fluid–accessible biomarker and druggable target in subarachnoid haemorrhage: Linking fatty acid dysregulation to microglial neuroinflammation
Source: Clin Transl Med. 2026 Jan 30;16(2):e70607. doi: 10.1002/ctm2.70607 (PMC12856223; doi:10.1002/ctm2.70607)
Supplement: Supplementary file 9 — Supporting Information [file CTM2-16-e70607-s002.docx]

**Table. S1. Basal characteristics of patients with SAH and controls.**

|  | *Cohort* | | |
| --- | --- | --- | --- |
|  | SAH (n=48) | Control (n=30) | *P*-value |
| Demographics |  |  |  |
| Age, year | 56 (47-64) | 37.5 (27-55) | <0.001* |
| Sex (Female) | 31 (64.5%) | 10 (33.3%) | 0.010* |
| Hunt-Hess high grade (3-5) | 20 (41.7%) | N/A | N/A |
| Comorbidities |  |  |  |
| Hypertension | 28 (58.3%) | 5 (16.7%) | <0.001* |
| Diabetes | 2 (4.2%) | 2 (6.7%) | 0.636 |
| Hypercholesterolemia | 2 (4.2%) | 2 (6.7%) | 0.636 |
| Location of aneurysm |  |  |  |
| Anterior circulation | 29 (60.4%) | N/A | N/A |
| Posterior circulation | 19 (39.6%) | N/A | N/A |
| Complications |  |  |  |
| DCI | 4 (8.3%) | N/A | N/A |
| Hydrocephalus | 3 (6.3%) | N/A | N/A |
| Functional outcomes |  |  |  |
| Unfavorable mRS (3-5) | 16 (33.3%) | N/A | N/A |

DCI, delayed cerebral ischemia; mRS, modified Rankin Scale
