# Supplementary material for: Adipocyte fatty acid‐binding protein as a cerebrospinal fluid–accessible biomarker and druggable target in subarachnoid haemorrhage: Linking fatty acid dysregulation to microglial neuroinflammation
Source: Clin Transl Med. 2026 Jan 30;16(2):e70607. doi: 10.1002/ctm2.70607 (PMC12856223; doi:10.1002/ctm2.70607)
Supplement: Supplementary file 10 — Supporting Information [file CTM2-16-e70607-s003.docx]

**Table S2.1** The primary antibodies used in the western blot.

| Primary antibodies | Origin | Dilution | Cat no. & Company |
| --- | --- | --- | --- |
| Anti-A-FABP | Goat, polyclonal | 0.25μg/mL | AF1443, R&D |
| Anti- Cleaved-Caspase-3 | Rabbit, polyclonal | 1:1000 | YC0006, Immunoway |
| Anti-Bax | Rabbit, polyclonal | 1:1000 | #50599, Proteintech |
| Anti- Bcl-2 | Rabbit, polyclonal | 1:1000 | #26593, Proteintech |
| Anti- TNF-α | Rabbit, polyclonal | 1:1000 | YT4689, Immunoway |
| Anti-IL-1β | Rabbit, polyclonal | 1:500 | YM4682, Immunoway |
| Anti-IL-6 | Rabbit, polyclonal | 1:1000 | GB11117, Servicebio |
| Anti-STAT3 | Rabbit, polyclonal | 1:1000 | T55292, Abmart |
| Anti- p-Stat3(Y705) | Rabbit, polyclonal | 1:1000 | T56566, Abmart |
| Anti-JAK1 | Rabbit, polyclonal | 1:1000 | T57173, Abmart |
| Anti-p-JAK1 (Tyr1022/Tyr1023) | Rabbit, polyclonal | 1:1000 | TA2012, Abmart |
| Anti-JAK2 | Rabbit, polyclonal | 1:1000 | T55287, Abmart, |
| Anti-p-JAK2 (Y1007+Y1008) | Rabbit, polyclonal | 1:1000 | T56570, Abmart |
| Anti-JAK3 | Rabbit, polyclonal | 1:1000 | PC2808, Abmart |
| Anti-JAK3(Tyr785) | Rabbit, polyclonal | 1:1000 | YP0756, Immunoway |
| Anti-CPT1A | Rabbit, polyclonal | 1:1000 | #15184, Proteintech |
| Anti-ACADL | Rabbit, polyclonal | 1:1000 | #17526, Proteintech |
| Anti-occludin | Rabbit, polyclonal | 1:1000 | TD7504, Abmart |
| Anti-β-actin | Mouse, Monoclonal | 1:5000 | #66009, Proteintech |
| Anti-β-tubulin | Rabbit, polyclonal | 1:10000 | AP0064, bioworld |

**Table S2.1** The primary antibodies used in the Immunofluorescence staining.

| Primary antibodies | Origin | Dilution | Cat no. & Company |
| --- | --- | --- | --- |
| Anti-A-FABP | Rabbit, monoclonal | 1:100 | ab92501, Abcam |
| Anti-IBA1 | Mouse, monoclonal | 1:100 | ab283319, Abcam |
| Anti-GFAP | Mouse, monoclonal | 1:100 | GB12096, Servicebio |
| Anti-NeuN | Mouse monoclonal | 1:100 | ab104224, Abcam |
| Anti-CD31 | Rabbit, monoclonal | 1:100 | ab76533, Abcam |
| Anti-IL-6 | Rabbit, polyclonal | 1:100 | GB11117, Servicebio |
| Anti-TNF-α | Rabbit, polyclonal | 1:100 | YT4689, Immunoway |
| Anti-ZO-1 | Rabbit, monoclonal | 1:100 | GB15195, Servicebio |
