# Supplementary material for: Adipocyte fatty acid‐binding protein as a cerebrospinal fluid–accessible biomarker and druggable target in subarachnoid haemorrhage: Linking fatty acid dysregulation to microglial neuroinflammation
Source: Clin Transl Med. 2026 Jan 30;16(2):e70607. doi: 10.1002/ctm2.70607 (PMC12856223; doi:10.1002/ctm2.70607)
Supplement: Supplementary file 11 — Supporting Information [file CTM2-16-e70607-s009.docx]

| **Table. S3 Primers (F, forward primer; R, reverse primer)** | | |
| --- | --- | --- |
| Application | Target gene | Sequences (5′-3′) |
| QPCR | β-actin (mouse) | F: TATGCTCTCCCTCACGCCATCC |
|  |  | R: GTCACGCACGATTTCCCTCTCAG |
|  | A-FABP (mouse) | F: TCACCGCAGACGACAGGAAG |
|  |  | R: AAGTCACGCCTTTCATAACACATTC |
|  | L-FABP (mouse) | F: ATGAACTTCTCCGGCAAGTACC |
|  |  | R: CTGACACCCCCTTGATGTCC |
|  | I-FABP (mouse) | F: GTGGAAAGTAGACCGGAACGA |
|  |  | R: CCATCCTGTGTGATTGTCAGTT |
|  | H-FABP (mouse) | F: ACCTGGAAGCTAGTGGACAG |
|  |  | R: TGATGGTAGTAGGCTTGGTCAT |
|  | E-FABP (mouse) | F: TGAAAGAGCTAGGAGTAGGACTG |
|  |  | R: CTCTCGGTTTTGACCGTGATG |
|  | iL-FABP (mouse) | F: CTTCCAGGAGACGTGATTGAAA |
|  |  | R: CCTCCGAAGTCTGGTGATAGTTG |
|  | B-FABP (mouse) | F: GGACACAATGCACATTCAAGAAC |
|  |  | R: CCGAACCACAGACTTACAGTTT |
|  | TNF-α (mouse) | F: CACGCTCTTCTGTCTACTGAACTTC |
|  |  | R: CTTGGTGGTTTGTGAGTGTGAGG |
|  | IL-1β(mouse) | F: GGTGTGT GACGTTCCCATTA |
|  |  | R: ATTGAGGTGGAGAGCTTTCAG |
|  | IL-6 (mouse) | F: CTTCTTGGGACTGATGCTGGTGAC |
|  |  | R: TCTGTTGGGAGTGGTATCCTCTGTG |
| PCR | WT(AP2WTS2) | F: CTTCTGAGGTGCACTCTATCCTC |
|  | KO(AP2KOS1) | F: TGCATCGCATTGTCTGAGTAGGTG |
|  | AP2(CONAS1) | R: AGGTCTTGTATGCCACAGCGGAC |
