# Supplementary material for: Adipocyte fatty acid‐binding protein as a cerebrospinal fluid–accessible biomarker and druggable target in subarachnoid haemorrhage: Linking fatty acid dysregulation to microglial neuroinflammation
Source: Clin Transl Med. 2026 Jan 30;16(2):e70607. doi: 10.1002/ctm2.70607 (PMC12856223; doi:10.1002/ctm2.70607)
Supplement: Supplementary file 12 — Supporting Information [file CTM2-16-e70607-s012.docx]

**Table. S4 Mortality rate and excluded numbers**

| **Groups** | **Mortality rate** | **Excluded** |
| --- | --- | --- |
| **Experiment 1** |  |  |
| Sham(n=12) | 0% (0/12) | 0 |
| SAH 3h(n=6) | 14.29% (1/7) | 0 |
| SAH 6h(n=3) | 25.00% (1/4) | 0 |
| SAH 12h(n=6) | 14.29% (1/7) | 0 |
| SAH 24h(n=12) | 18.75% (3/16) | 1 |
| SAH 36h(n=3) | 0% (0/3) | 0 |
| SAH 48h(n=6) | 0% (0/7) | 1 |
| SAH 72h(n=6) | 0% (0/6) | 0 |
| SAH 5D(n=3) | 0% (0/3) | 0 |
| Total (n=57) | 9.23% (6/65) | 2 |
| **Experiment 2-6** |  |  |
| Sham (n=38) | 0% (0/38) | 0 |
| SAH+ Vehicle (n=38) | 9.00% (4/44) | 2 |
| SAH+BMS309403 (n=38) | 10.87% (5/46) | 3 |
| SAH WT (n=38) | 6.98% (3/43) | 2 |
| SAH KO (n=38) | 11.11% (5/45) | 2 |
| Total (n=190) | 6.16% (17/216) | 9 |
| **Experiment 7** |  |  |
| SAH KO+CA1 (n=12) | 13.33% (2/15) | 1 |
